# Resembling Graphene/Polymer Aerogel Morphology for Advancing the CO_2_/N_2_ Selectivity of the Postcombustion CO_2_ Capture Process

**DOI:** 10.1021/acs.iecr.3c02989

**Published:** 2024-04-09

**Authors:** Iranzu Barbarin, Monika Fidanchevska, Nikolaos Politakos, Luis Serrano-Cantador, Juan Antonio Cecilia, Dolores Martín, Oihane Sanz, Radmila Tomovska

**Affiliations:** † POLYMAT and Department of Applied Chemistry, University of the Basque Country UPV/EHU, 20018 Donostia-San Sebastián, Spain; ‡ Biopren Group, Inorganic Chemistry and Chemical Engineering Department, Nanochemistry University Institute (IUNAN), Universidad de Córdoba, 14014 Córdoba, Spain; § Inorganic Chemistry, Crystallography and Mineralogy, University of Málaga, 29071 Málaga, Spain; ∥ Macrobehaviour-Mesostructure-Nanotechnology SGIker Service, Faculty of Engineering of Gipuzkoa, University of the Basque Country (UPV/EHU), Plaza Europa 1, 20018 Donostia-San Sebastian, Spain; ⊥ Department of Applied Chemistry, University of the Basque Country, 20018 Donostia-San Sebastián, Spain; # Ikerbasque, Basque Foundation for Science, Maria Diaz de Haro 3, 48013 Bilbao, Spain

## Abstract

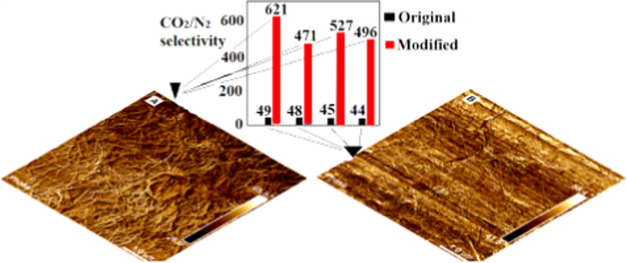

The separation of CO_2_ from N_2_ remains a highly challenging task in postcombustion CO_2_ capture processes, primarily due to the relatively low CO_2_ content (3–15%) compared to that of N_2_ (70%). This challenge is particularly prominent for carbon-based adsorbents that exhibit relatively low selectivity. In this study, we present a successfully implemented strategy to enhance the selectivity of composite aerogels made of reduced graphene oxide (rGO) and functionalized polymer particles. Considering that the CO_2_/N_2_ selectivity of the aerogels is affected on the one hand by the surface chemistry (offering more sites for CO_2_ capture) and fine-tuned microporosity (offering molecular sieve effect), both of these parameters were affected in situ during the synthesis process. The resulting aerogels exhibit improved CO_2_ adsorption capacity and a significant reduction in N_2_ adsorption at a temperature of 25 °C and 1 atm, leading to a more than 10-fold increase in selectivity compared to the reference material. This achievement represents the highest selectivity reported thus far for carbon-based adsorbents. Detailed characterization of the aerogel surfaces has revealed an increase in the quantity of surface oxygen functional groups, as well as an augmentation in the fractions of micropores (<2 nm) and small mesopores (<5 nm) as a result of the modified synthesis methodology. Additionally, it was found that the surface morphology of the aerogels has undergone important changes. The reference materials feature a surface rich in curved wrinkles with an approximate diameter of 100 nm, resulting in a selectivity range of 50–100. In contrast, the novel aerogels exhibit a higher degree of oxidation, rendering them stiffer and less elastic, resembling crumpled paper morphology. This transformation, along with the improved functionalization and augmented microporosity in the altered aerogels, has rendered the aerogels almost completely N_2_-phobic, with selectivity values ranging from 470 to 621. This finding provides experimental evidence for the theoretically predicted relationship between the elasticity of graphene-based adsorbents and their CO_2_/N_2_ selectivity performance. It introduces a new perspective on the issue of N_2_-phobicity. The outstanding performance achieved, including a CO_2_ adsorption capacity of nearly 2 mmol/g and the highest selectivity of 620, positions these composites as highly promising materials in the field of carbon capture and sequestration (CCS) postcombustion technology.

## Introduction

The increase in anthropogenic greenhouse gas emissions in the atmosphere has steadily increased since the Industrial Revolution. The continued global demand for energy heavily relies on the combustion of fossil fuels, making power plants the primary source of carbon dioxide (CO_2_) emissions into the atmosphere.^1^ The accumulation of greenhouse gases, particularly CO_2_, significantly disrupts the Earth’s climate balance. The critical level of CO_2_ concentration, currently just above 400 ppm, is closely linked to global warming and climate change.^2^ To address the increasing energy requirements and the limited maturity of renewable energy sources, carbon capture and sequestration (CCS) technology has emerged as a crucial strategy to enable the utilization of fossil fuels while mitigating CO_2_ emissions directly from the source.^3^ However, a major challenge in postcombustion capture lies in the low CO_2_ concentration relative to N_2_ in the exhaust gas, which impacts capture efficiency and requires a high selectivity in the separation process.^4,5^

Currently, the widely employed industrial method for selective CO_2_ gas capture is the scrubbing process using monoethanolamine (MEA) solvent.^6,7^ However, to overcome the limitations associated with the harsh nature of the solvent and the significant heat requirement for sorbent regeneration, various new techniques and materials have been proposed as alternative solutions in the past decade,^8^ with some already implemented. Among these, physical or chemical adsorption using advanced solid porous sorbents has emerged as a leading method. This approach relies on the selective adherence of CO_2_ molecules to the solid surface through a process that is influenced by both the characteristics of the adsorbate (molecular size and polarity) and the adsorbent (pore size and polarity).^9^ Inorganic adsorbents (such as zeolites), carbon-based materials, porous polymers, and calcium oxides are among the key materials utilized in this process.^10,11^ Apart from high adsorption capacities and selectivity, practical application of adsorbents requires attributes such as high thermal stability, mechanical strength, water stability, corrosion resistance, and stability during cyclic operations. While significant CO_2_ adsorption capacity is a desirable parameter, equally or even more important is the high selectivity of the material to adsorb CO_2_ over N_2_, O_2_, or CH_4_.^12^

The separation of CO_2_ from N_2_ is of utmost importance but remains highly challenging, particularly in postcombustion flue gas mixtures where CO_2_ typically constitutes 3–15%, while N_2_ makes up more than 70%.^13^ While significant efforts have been devoted to enhancing CO_2_ adsorption capacity, the investigation of selectivity has often been treated as a secondary concern, becoming a current bottleneck in practical applications,^14^ because many porous adsorbents exhibit low CO_2_ selectivity compared to other gases such as N_2_, O_2_, and H_2_O.^15,16^ In the quest for adsorbents that can facilitate cost-efficient carbon capture technology under postcombustion conditions, a dilemma arises between adsorption capacity and CO_2_/N_2_ selectivity, both of which have a significant impact on process costs. Recent research by Willey et al. has shown that the costs of CO_2_ capture per ton can be influenced more by selectivity than by adsorption capacity. They demonstrated that increasing the adsorption capacity from 1 to 4 mmol/g led to a reduction in CCS costs by approximately 5 US$/t, whereas augmenting the CO_2_/N_2_ selectivity from 50 to 500 resulted in a cost reduction of 12 US$/t.^17^ Higher selectivity leads to an increased purity of the captured gas, irrespective of the regeneration pressure ratio. Therefore, increasing selectivity over N_2_ represents a potential strategy to enhance the economic viability of CO_2_ capture in postcombustion mode.

Different strategies have been proposed to enhance the selectivity of CO_2_ over N_2_, with a focus on increasing the CO_2_ adsorption capacity by creating “CO_2_-philic” spots. CO_2_-philic materials, similar to hydrophilic materials, exhibit a high affinity toward CO_2_ due to their large surface areas, functional groups containing heteroatoms (such as oxygen, sulfur, or nitrogen), specific polarity, and/or basic character. In the context of adsorption onto carbon-based materials, which are of interest in this study, the adsorption of gases is primarily governed by van der Waals forces. Therefore, the most crucial strategy for improving adsorption is to increase the available surface area by controlling the textural properties of the adsorbent. Additionally, fine-tuning the micropores and small mesopores to have diameters smaller than the kinetic diameter of nitrogen molecules and larger than that of CO_2_ can lead to the creation of adsorbents that preferentially interact with CO_2_ rather than N_2_ gas.^14^ On the other hand, incorporating heteroatom-containing functionalities into carbon-based adsorbents, either through doping or surface modification, can enhance the electrostatic interaction with CO_2_ molecules.^18−25^ Nitrogen-containing functionalities, which provide basicity to the surface, promote Lewis acid–base interactions,^26^ while hydroxyl and carbonyl groups establish strong interactions with CO_2_ due to their higher electron densities.^27^ Although higher CO_2_ adsorption capacities have been achieved in many cases, a higher selectivity performance is not always guaranteed. Conversely, achieving lower N_2_ adsorption coupled with higher CO_2_ adsorption can be a powerful approach toward CO_2_/N_2_ selective carbon capture and storage (CCS) technology.

Some theoretical and experimental studies have explored the concept of “N_2_-phobic” materials, which naturally repel N_2_ molecules, highlighting the importance of achieving high selectivity.^28−30^ According to these reports, N_2_-phobic materials should possess a hierarchical pore structure with well-defined small mesopores that have diameters smaller than the kinetic diameter of nitrogen but larger than that of CO_2_. This structural configuration disrupts and reduces the level of N_2_ adsorption. In terms of surface chemistry, there are reports suggesting that azo-bridge polymer chains can exhibit N_2_-phobic characteristics, with selectivity as high as 300 obtained at a temperature of 323 K.^31^

Among solid sorbents, three-dimensional (3D) graphene-based aerogels attract attention due to their interconnected networks that exhibit large accessible specific surface areas and hierarchical porous structures rich in small mesopores, which provide high and fast CO_2_ adsorption from flue gas.^32,33^ There are different strategies for improvement of the selective CO_2_ physisorption by 3D graphene-based structures, which are based on thermodynamic and kinetic principles, e.g., CO_2_ adsorption by physical interactions or by the molecular sieving effect.^34−36^ In porous graphene-based materials, the control of purely monomodal pore size is quite difficult.^37^ In our previous works, 3D monolithic structures were obtained by chemical reduction of graphene oxide (GO) in aqueous dispersion, where the reduced graphene oxide (rGO) layers were joined together by a self-assembly hierarchical process. By varying the reaction parameters, control of the pore size distribution was achieved, resulting in more than 70% mesopore-rich structures.^38^ Moreover, the polarizability of graphene-based materials could be achieved by a straightforward process, by the addition of functional polymer particles, as can be seen in our previous work.^39^ CO_2_-philic groups could enhance physical interactions over the CO_2_ molecule with respect to N_2_. On the other hand, the ubiquitous presence of oxygen functionalities onto GO increases the interactions with CO_2_ molecules because these electron-rich functionalities bind with the carbon of CO_2_.^40^ As reported in our previous works, our 3D graphene–polymer materials show adsorption capacities that vary between 0.5 and4 mmol/g and selectivity values between 50 and 90.^38−40^ These values are sufficiently high to meet the requirements needed to be applied as adsorbent materials in postcombustion capture. Nevertheless, to further decrease the CO_2_ capture costs, it is of utmost importance to raise the CO_2_/N_2_ selectivity.

In order to make our carbon-capturing technology based on self-assembly graphene closer to practical application, in the present work, we altered the synthesis procedure toward augmentation of the fraction of oxygen functionalities and the content of the small mesopores within the monolithic structure. The achieved impact over CO_2_/N_2_ selectivity was huge, resulting in a rise of the selectivity for more than 1 order of magnitude with respect to reference monoliths, achieving selectivity in a range of 470–620. To the best of the authors’ knowledge, this is far higher CO_2_/N_2_ selectivity reported for carbon-based adsorbents,^20,41−44^ which is 5-fold the maximum reported by Chowdhury et al.^45^ A short review of the highest CO_2_/N_2_ selectivity reported so far is shown in Table 1.

**Table 1:** Short Review on CO_2_/N_2_ Selectivity of Carbon-Based Adsorbents with Their Textural Properties

refs	*S*_BET_ (m^2^/g)	CO_2_ capacity (1 atm, 298 K), mmol/g	selectivity CO_2_/N_2_ (0.15/0.85), 298 K
(20)	776	4.12	102
(20)	1930	5.18	153
(41)	940	3.5	29
(42)		4.3	34
(43)	484	2.19	43
(44)	497	1.4	70
(45)	1316	1.06	162

Different from all published discussions on CO_2_/N_2_ selectivity, our results have demonstrated that the surface chemistry and textural properties are not the only decisive for N_2_-phobicity. The attempts to reveal the outstanding CO_2_/N_2_ selectivity achieved in this work have shown that the drop in N_2_ adsorption was induced by a change in the surface morphology. Namely, while attempting to make the aerogel more porous, we turned its surface to be more densely oxidized and subsequently stiffer and crumpled. The results have shown that this type of surface is highly N_2_-phobic. Therefore, this work contributes to a better understanding of the N_2_-phobic context of carbon-based porous materials and can be an excellent guide on how to improve the selectivity in the graphene/polymer porous materials and to reduce the cost of the postcombustion CCS.

## Materials and Methods

### Materials

The following materials were used throughout this study: deionized water, graphene oxide (GO) aqueous dispersion containing more than 95% monolayer GO with a concentration of 4 mg/mL (Graphenea), l-ascorbic acid (AsA, ≥99%, Sigma-Aldrich), methyl methacrylate (MMA, Quimidroga), 2-acrylamido-2-methyl-1-propanesulfonic acid (AMPS, 99%, Sigma-Aldrich), sodium 4-vinylbenzensulfonate (NaSS, ≥90%, Sigma-Aldrich), Dowfax 2A1 (Dow Chemical Company), potassium persulfate (KPS, ≥99%, Sigma-Aldrich), and *tert*-butyl hydroperoxide solution (TBHP, 70 wt % in H_2_O, Sigma-Aldrich).

### Synthesis of 3D Reduced Graphene Oxide Aerogels

**Figure 1:**
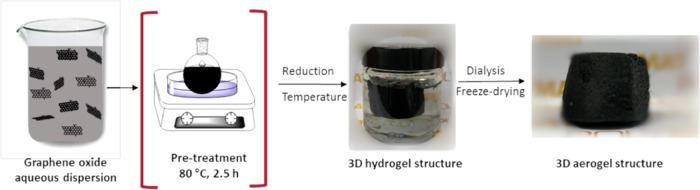
Schematic illustration of the synthesis of a 3D rGO monolithic structure.

The aerogels were synthesized in a few steps: (i) GO aqueous dispersion is sonicated for 1 h at 25 °C (amplitude of 70% and energy pulsed at 0.5 Hz). A Hielscher Sonicator-UIS250v, Hielscher Ultrasonics GmbH, Teltow, Germany was used for that aim. (ii) Two different routes were followed afterward, in one of which the GO dispersion was pretreated at 80 °C for 2.5 h, and in the other, this step was skipped. (iii) For reduction of GO, AsA was added in both treated and nontreated dispersions, followed by stirring for 0.5 h at ambient temperature. The reduction reaction was performed at different temperatures: 45, 60, and 90 °C. Three-dimensional reduced graphene oxide (rGO) monolithic hydrogels were created in this step. (iv) Purification of hydrogels was performed by a dialysis process with deionized water, changing it daily until the water conductivity values were lower than 10 μS/cm (on average, 1 week was needed to achieve it). (v) The last step is drying hydrogels and turning them into aerogels by a freeze-drying technique using a Telstar LyoQuest 55 at −49 °C and 0.2 mbar. The duration of the drying process was 3 days. In Figure 1, the synthesis of the 3D rGO monolithic structures is schematically presented.

### Synthesis of Polymer Particles

The synthesis procedure is explained elsewhere.^40^ Two types of MMA functional polymer particles dispersed in water were synthesized. Shortly, the synthesis was performed by a seeded semicontinuous emulsion polymerization process. Two different functional monomers were copolymerized with the main monomer MMA, either NaSS or AMPS (chemical structures are presented in Figure S1 in the Supporting Information) in an amount of 1 wt % based on MMA. The reactions were carried out under a nitrogen atmosphere in a glass reactor fitted with a stainless steel stirrer, a reflux condenser, a thermocouple, a sampling tube, and a feeding inlet. The temperature in the reactor was controlled by automatic control software (Camile TG, Biotage). Table S1 presents details on the employed formulations.

As a result, two colloidally stable polymer particle dispersions were obtained with 30 wt % solid contents, one of them made of MMA polymer particles functionalized with sulfonate functionality and the other made of MMA particles functionalized with amine moieties. ^1^H NMR was used to measure the incorporations of both nonvolatile monomers NaSS and AMPS, showing that NaSS was incorporated only 37% onto MMA particles, whereas almost all added AMPS was incorporated (100%). The conversion of the main monomer MMA was followed gravimetrically and in both cases was almost complete.

### Synthesis of rGO/Polymer Composite Aerogels

**Figure 2:**
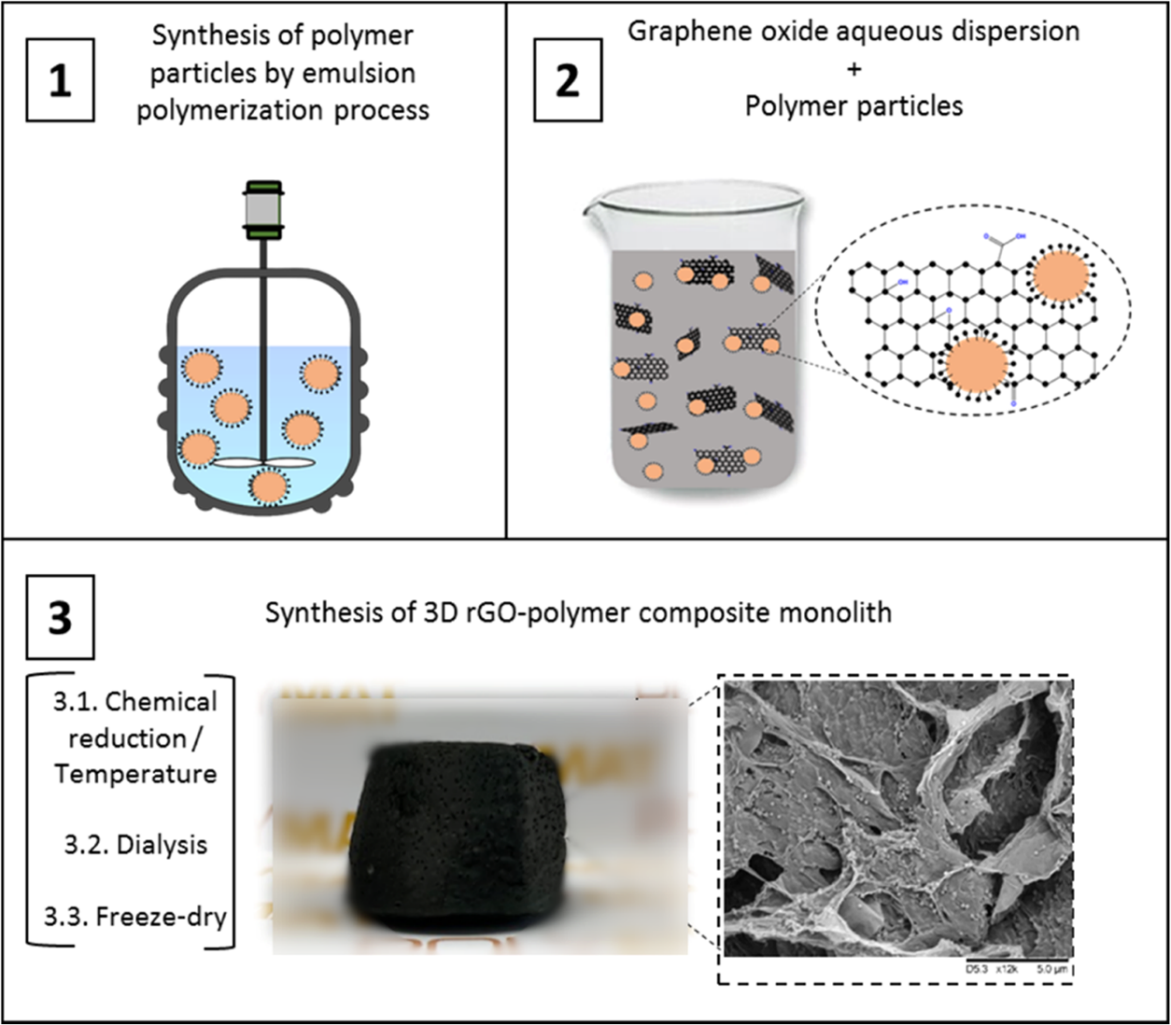
Schematic illustration of the synthesis of a 3D rGO–polymer monolithic structure.

rGO–polymer composite aerogels were synthesized by the same procedure as the neat rGO structure. Prior to the reduction process, both aqueous dispersions of GO and MMA particles were mixed for 2 h at room temperature (RT). Polymer particles adsorbed onto GO platelets, as it is explained in the Supporting Information. Afterward, AsA was added to the composite dispersions (GO/AsA 1:1 weight ratio) and stirred for 0.5 h. Then, the dispersions were placed in an oven at different temperatures overnight (45, 60, and 90 °C) to induce the reduction process, producing composite monolithic hydrogel structures. All of the quantity of the polymer used was incorporated in the structures, as confirmed by gravimetrical analysis of the residual water after formation of the monolith. Subsequently, the hydrogel was cleaned and dried similarly to the neat rGO monoliths, as explained previously. The straightforward experimental procedure of the 3D monolithic composed of reduced graphene oxide and polymer particles is schematically described in Figure 2.

### Characterization of 3D Monoliths

Thermal stability and the estimation of the amount of residual oxygen-containing functionalities within the 3D graphene structure were conducted by thermogravimetric analyses in a TGA/DSC 3 + apparatus (Mettler Toledo). Samples of about 2 mg were heated under a N_2_ atmosphere (90 mL/min) from 25 to 800 °C at a rate of 10 °C/min.

Textural properties of the monoliths were examined by means of N_2_ adsorption–desorption measurements performed at −196 °C in a Micromeritics ASAP 2020 apparatus. Before the analysis, the materials were degassed at 100 °C for 8 h under vacuum. From N_2_ adsorption–desorption isotherms, the specific surface area (*S*_BET_) was determined from the Brunauer–Emmett–Teller (BET) equation. Furthermore, the *t*-plot method was used to evaluate the micropore volume (*V*_micro_). Finally, the pore volume (*V*_total_) and pore size distribution were calculated using the method proposed by Barrett–Joyner–Halenda (BJH method).

The surface morphology of the monolithic structures was analyzed using scanning electron microscopy (SEM, Hitachi TM3030 tabletop model) at an accelerating voltage of 15 kV. The samples were coated with a thin gold layer prior to analysis.

X-ray photoelectron spectroscopy (XPS) was used to study the surface chemical states of the composing elements of monoliths. The measurements were performed in a SPECS system (Berlin, Germany) equipped with a Phoibos 150 1D-DLD analyzer and monochromatic radiation source Al Kα (1486.7 eV). The spectrometer was previously calibrated with Ag (Ag 3d_5/2_, 368.26 eV).

The surface roughness of such rGO layers was analyzed by means of an atomic force microscope (AFM, Dimension ICON from Bruker), using an AFM-based tapping technique with a resonant frequency of 320 kHz and a spring constant of 37 N/m. Prior to the analysis, GO aqueous dispersion with a concentration of 0.01 mg/mL was placed into an ultrasonic bath for 15 min at 25 °C, a function of 45 kHz, and at 70% power conditions (Fisher, Bioblock Scientific). Both of them were then reduced by AsA (GO/AsA 1:1) for 30 min at RT and finally were drop-cast in a silicon wafer (4″ silicon wafer, TED PELLA, INC.) substrate.

Initially, an appropriate amount (25 mL) of GO dispersion was diluted (with 50 mL of water). Then, an appropriate quantity (0.1 g) of AsA was added (GO/AsA = 1:1). After that, one of the samples was submitted to the pretreatment step and the other without it. Pretreatment was performed in an oven at 80 °C for 2 h. Finally, both mixtures of GO/AsA (pre- and non-pretreated) were transferred into a reactor to be reduced at a given temperature (45 and 90 °C) for 30 min with agitation of 124 rpm.

N_2_ and CO_2_ adsorption isotherms were measured by using a Micromeritics ASAP 2020 Analyzer (i.e., volumetrically) at 25 °C and 1 atm. Prior to analysis, samples were outgassed at 110 °C and 10^–4^ mbar for 8 h.

The adsorption selectivity of CO_2_ over N_2_ was calculated according to a simplified Ideal Adsorption Solution Theory (IAST) taking into account the data for the pure-component adsorption equilibria at the same temperature (25 °C) and using the same monolithic adsorbent.^46^ The individual isotherms were modeled by Langmuir, Freundlich, and their combined model isotherms. The viability of these models was evaluated by the correlation coefficient (*R*^2^). The best fitting toward the specific isotherm model implies an *R*^2^ closer to unity, and in this study, Freundlich gave the highest *R*^2^. The linear form of the Freundlich model used is presented by eq 1
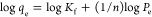
1where *q*_e_ is the amount of gas adsorbed (cm^3^/g); *P*_e_ is the equilibrium pressure (bar); and *K*_f_ (cm^3^/g bar^1/*n*^) and *n* are Freundlich constants.

The selectivity was calculated according to eq 2
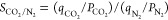
2where *P*_i_ is the partial pressure of the i component (CO_2_ or N_2_) and *q*_i_ is the amount of CO_2_ or N_2_ adsorbed (cm^3^/g).

The partial pressures of both N_2_ and CO_2_ are those for flue gas from a coal-fired power plant, containing approximately 85% N_2_ and 15% CO_2_.

## Results and Discussion

The formation mechanism of the aerogel structures was explained in our previous works.^38,39^ Shortly, the synthesis consists of reaction-induced self-assembly of rGO platelets in aqueous dispersion. For composite aerogels, polymer particle aqueous dispersion is added to the GO dispersion prior to reduction. Initially, GO platelets are amphiphilic and form a colloidal dispersion in water. After reduction, their hydrophobicity increased substantially, inducing their incomplete aggregation and formation of a monolithic structure, in which all of the solids present in the dispersions (including polymer particles) are incorporated (Figure 2). The monolithic structures are swelled with water, forming a kind of hydrogel, which after freeze-drying gave rise to hydrophobic, highly porous, 3D monolithic aerogels made either of neat rGO or rGO/polymer composites. The driving force for this process is the sudden increase in the surface energy in the dispersion after GO reduction. In our previous works,^38−40^ prior to the reduction, the GO dispersions are subjected to 80 °C pretreatment with the aim of homogenization of the dispersions. Nevertheless, it was found that there is a loss of oxygen functionalities and partial rGO restacking, which increases the hydrophobicity of the initial GO platelets and lowers the driving force for the monolith formation. In this work, we avoided this pretreatment, expecting to create a higher driving force and subsequently more compact aerogels with altered surface chemistry and textural properties. Namely, aerogels richer in residual oxygen functionalities and small mesopores are expected, and subsequently with improved performance for selective CO_2_ capture. Moreover, as the procedure is shorter and less energy consuming, the carbon footprint of the synthesis process would decrease further.

The reduction process was performed at different reduction temperatures (45, 60, and 90 °C), at a constant GO/AsA mass ratio of 1:1, in two different procedures, creating six neat rGO aerogels. In Table 2, the aerogels’ characteristics are shown. In the aerogels’ nomenclature, the first number refers to the reduction temperature and the last to the pretreatment temperature.

**Table 2:** Amount of the Residual Oxygen-Containing Functional Groups and Textural Properties of the 3D rGO Structures

material	% O-functionality	*S*_BET_ (m^2^/g)	*V*_total_ (cm^3^/g)	*V*_micro_ (cm^3^/g)	% Micro
45_Blank_80	14	160	0.29	0.01	2.7
45_Blank	17	137	0.16	0.02	13.3
60_Blank_80	9	172	0.65	0.001<	0.2
60_Blank	12	146	0.24	0.01	2.9
90_Blank_80	3	299	1.36	0.001<	0.1
90_Blank	5	214	0.35	0.02	4.3

**Figure 3:**
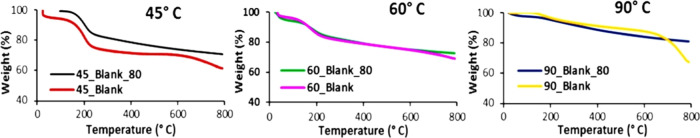
TGA analysis of neat rGO aerogels synthesized at 40, 60, and 90 °C with 80 °C treatment and without it.

The thermal stability and amount of residual oxygen functional groups were determined by TGA analysis. The TGA graphs of the neat rGO structures are presented in Figure 3. The weight loss of all curves occurred in three steps, in which the first weight decay until 100 °C is related to the humidity, the second weight drop between 100 and 225 °C corresponds to the loss of the residual oxygen functionalities, and the last loss is assigned to the graphenic structure. According to Figure 3, by increasing the reduction temperature, more compact and less functionalized structures were formed due to a faster reduction and self-assembly process.^38,39^ From the TGA curves, the weight loss occurring in a range of 100–225 °C was considered to be a fraction of oxygen-containing functional groups, and it is shown in Table 2. This fraction decreases in the aerogels produced at higher reduction temperatures. On the other hand, when materials were pretreated at 80 °C prior to the reduction process, the fraction of oxygen functionalities was much lower than those in the respective materials produced avoiding the pretreatment, e.g., 14 versus 17% for 45 °C reduction temperature. This difference is smaller for aerogels produced at higher temperatures. This confirms the hypothesis that by avoiding the pretreatment, a denser functionalization of rGO platelets within the resulting aerogels would be achieved.

**Figure 4:**
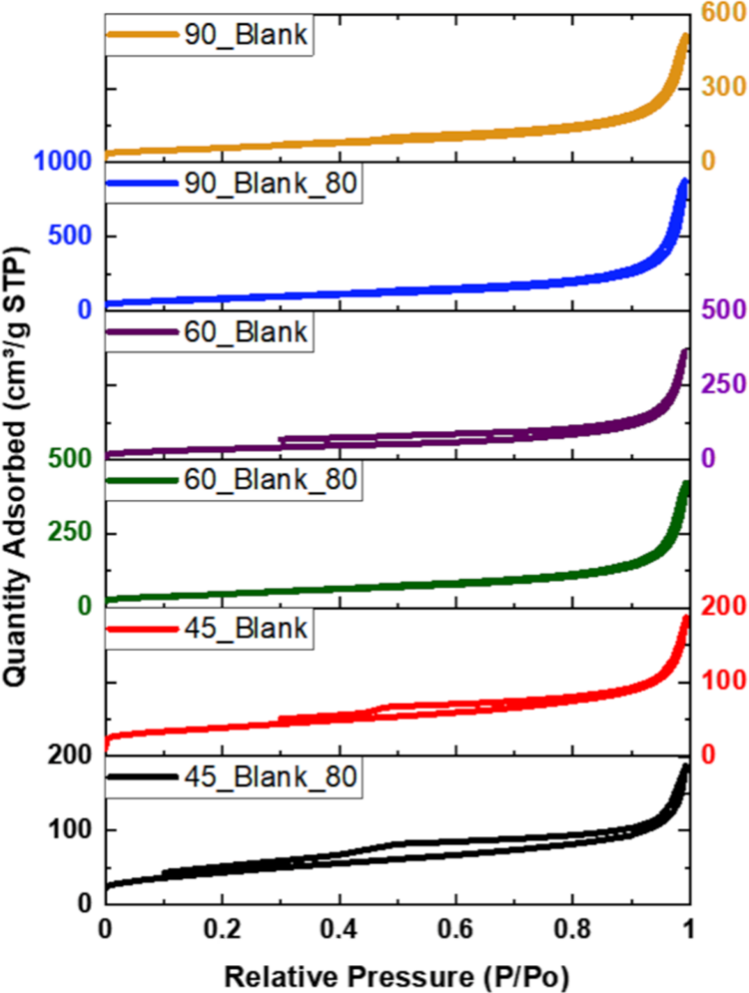
N_2_ adsorption–desorption isotherms of neat rGO aerogels produced at 45, 60, and 90 °C with and without 80 °C pretreatment.

N_2_ adsorption–desorption isotherms of all neat rGO aerogels are shown in Figure 4. All isotherms are of type IV, typical for mesoporous materials.^47^ It might be observed that there is a small hysteresis that appears, indicating that the capillary condensation phenomenon is happening. The textural properties determined from the isotherms shown in Figure 4 are placed in Table 2. According to Table 2, the aerogels produced at higher temperatures present larger BET specific surface areas and total volume of the pores, reaching a maximum surface area of 299 m^2^/g and pore volume of 1.359 cm^3^/g (90_Blank_80). The pretreated platelets allowed the development of higher surface area and total pore volume, with a negligible fraction of micropores, whereas the aerogels obtained without pretreatment are less porous, with a higher fraction of micropores. Likely, the more oxidized surface of the nontreated platelets prevents their complete restacking during the self-assembly, resulting in information on the higher fraction of micropores. On the other hand, the higher BET area of the pretreated aerogels is likely to result in the initial restacking of the GO platelets occurring during the 80 °C pretreatment, giving rise to a large number of small mesopores. Considering that without pretreatment, a higher fraction of oxygen functionalities is distributed throughout the lower available BET area, these aerogels are much more densely functionalized. According to our previous experience, such characteristics of the monoliths have shown to be favorable for the selective CO_2_/N_2_ capture at ambient conditions (1 bar and 25 °C), at which the interplay between the BET surface area and the fraction of oxygen functionalities is decisive, while the textural characteristics did not affect it significantly.^38−40^

**Figure 5:**
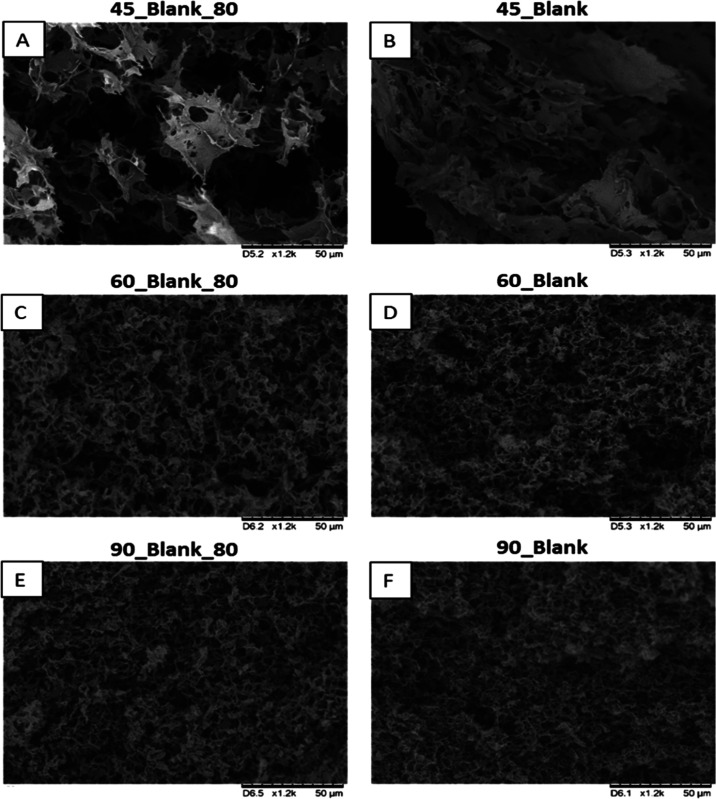
SEM images of 45_Blank_80 (A), 45_Blank (B), 60_Blank_80 (C), 60_Blank (D), 90_Blank_80 (E), and 90_Blank (F) on a 50 μm bar scale.

The structure and morphology of the aerogels were characterized by SEM, and the images are gathered in Figure 5. All aerogels present a porous structure, which is more compact and with a higher number of lower-size pores for higher reduction temperatures, as shown in SEM images in Figure 5 (90_Blank_80 in Figure 5E and 90_Blank in Figure 5F). Therefore, these aerogels present a higher porosity in concordance with the larger BET surface areas (Table 2). In fact, materials formed at 90 °C present a smaller overall volume than those obtained at milder temperatures for the same quantity of material.^38^ On the other hand, there are no important differences in the SEM images between the structures synthesized with or without pretreatment.

**Figure 6:**
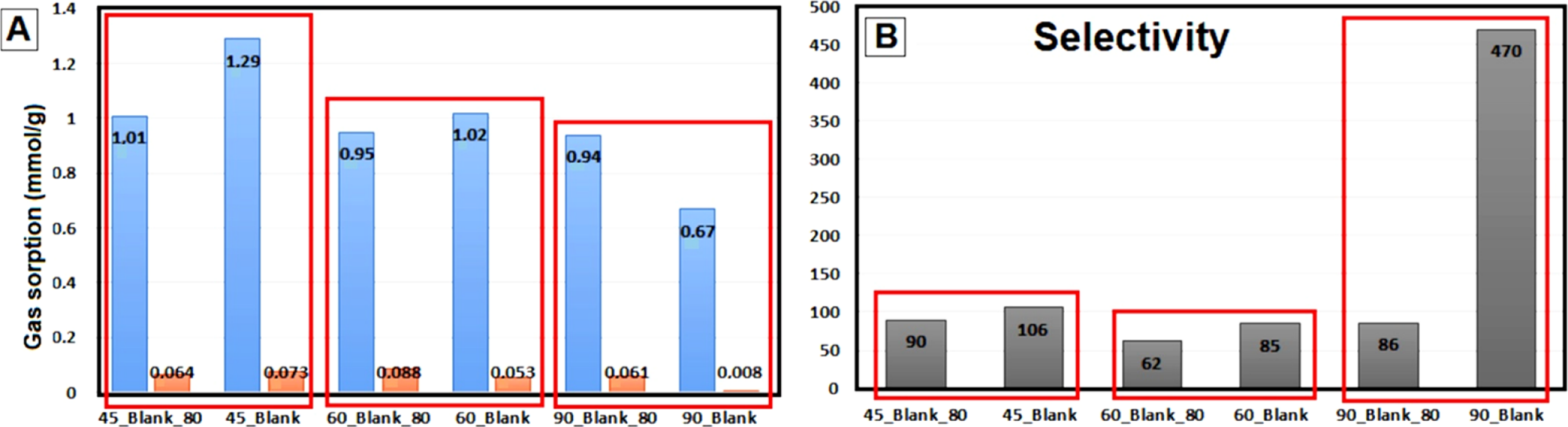
Comparison of CO_2_ and N_2_ adsorption (A) and CO_2_/N_2_ selectivity (B) of neat rGO monoliths produced at 45, 60, and 90 °C with and without pretreatment.

In Table S2 in the Supporting Information and in Figure 6, the CO_2_ and N_2_ adsorption capacities at 1 atm and 25 °C and IAST selectivity for each of these monoliths are presented. It is worth mentioning that the IAST method for calculation of the CO_2_/N_2_ selectivity on the basis of low-pressure single gas adsorption isotherms was utilized, besides the important limitation of the same. Its simplicity, robustness, and wide application make it a useful tool to compare the performance of the materials reported in the literature. For that aim, the Freundlich isotherm (eq 1) was used to model the equilibrium adsorption data, as it shows the best fitting of the experimental adsorption data of both gases CO_2_ and N_2_.

The individual adsorption–desorption isotherms obtained at 25 °C for CO_2_ and N_2_ are presented in Figure S2. The best fitting of these experimental results was obtained with the Freundlich isotherm (eq 1, Materials and Methods Section), and the fitting curves for CO_2_ and N_2_ and the parameters in the Freundlich isotherm are presented in Figure S3 in the Supporting Information. The selectivity calculated by eq 2 (Materials and Methods Section) is presented in Figure 6B.

Figure 6A reveals that the neat rGO aerogels synthesized at 45 and 60 °C by avoiding the pretreatment adsorbed a higher CO_2_ quantity, while that of N_2_ was not affected the same for the pretreated aerogels. Consequently, improved selectivity was attained, as shown in Figure 6B. Nevertheless, the aerogel produced at 90 °C without the pretreatment (90_Blank) presents lower adsorption of both CO_2_ and N_2_. Likely, the CO_2_ adsorption drop is a consequence of the important decrease of the surface area when the pretreatment was avoided (Table 2) without the important change of the oxygen functionality fraction. However, this aerogel has shown to be almost completely N_2_-phobic, which results in amplified CO_2_/N_2_ selectivity to 470 from 86 when the pretreatment was avoided. This result confirms the findings obtained from the theoretical studies of the selectivity in C-based porous materials, which claimed that excellent selectivity could not be attained only by improving the CO_2_ adsorption capacity, but N_2_-phobicity has to be gained, too.^28,29^Table 1 shows reported results on the CO_2_/N_2_ selectivity for similar carbon-based materials, which are at least a third of the presented value.

Our previous studies have shown that the neat rGO structures are not stable and lost mass in cyclic operations, which was resolved by addition of functionalized polymer particles (10–40 wt %) into the structures.^38,39^ In order to study how the addition of functionalized polymer particles affects the textural properties and adsorption characteristics when the pretreatment was avoided, herein, a portfolio of 3D graphene–polymer aerogels were synthesized at 45 and 90 °C without the 80 °C pretreatment of GO prior to reduction. For that aim, NaSS-functionalized and AMPS-functionalized MMA polymer particle dispersions were employed to produce the composites. MMA polymer particles were selected due to their high *T*_g_ (about 105 °C),^48^ which ensures that during the drying process, the particles would keep the particle morphology, avoiding covering the rGO surface. It is important because our previous results (experimental and theoretical studies) have shown that the CO_2_ molecules have a higher affinity toward the neat rGO surfaces than toward the polymers, even though the composite structures may achieve higher capture and selectivity.^38−40,49^ A small quantity of the NaSS monomer used for the synthesis of polymer particles introduced sulfonate functional groups onto the MMA particle surface, whereas that of AMPS introduced both sulfonic acid and amide moieties (chemical structures of both functional monomers are shown in Figure S1). Furthermore, two different amounts of polymer particles were used (10 and 40 wt %) based on the GO quantity, and the aerogels were produced at two reduction temperatures (45 and 90 °C) with a constant amount of the reducing agent (GO/AsA mass ratio of 1:1). Table 3 presents the characteristics of the 3D rGO–polymer composite aerogels, which were compared with those of the counterpart aerogels synthesized at 45 and 90 °C from the pretreated GO, as reported recently.^40^ For easier comparison, the characteristics of the pretreated aerogels already reported are shown in parentheses in Table 3. In the nomenclature of the composite monoliths, the first number refers to the reduction temperature, followed by the functional monomer used, and finished with the polymer fraction.

**Table 3:** Amount of the Residual Oxygen-Containing Functional Groups and Textural Properties of 3D rGO–Polymer Monolithic Structures Synthesized by Avoiding the Pretreatment of GO Dispersion at 80 °C Prior to Reduction^a^^40^

material^b^	% O-functionality	*S*_BET_ (m^2^/g)	*V*_total_ (cm^3^/g)	*V*_micro_ (cm^3^/g)	% Micro
45_NaSS_10	24 (12.8)	147 (185)	0.151 (0.184)	0.026 (0.035)	17.1 (18.8)
45_NaSS_40	19 (10.7)	98 (143)	0.150 (0.176)	0.011 (0.019)	7.1 (10.9)
45_AMPS_10	24 (12.9)	80 (170)	0.114 (0.170)	0.013 (0.032)	11.8 (19.1)
45_AMPS_40	16 (10.6)	112 (118)	0.108 (0.152)	0.021 (0.016)	19.6 (10.5)
90_NaSS_10	2 (3.6)	246 (199)	0.426 (0.337)	0.012 (0.016)	2.9 (4.9)
90_NaSS_40	5 (3.2)	181 (177)	0.324 (0.313)	0.012 (0.011)	3.6 (3.6)
90_AMPS_10	6 (3.8)	210 (207)	0.360 (0.348)	0.014 (0.016)	3.9 (4.6)
90_AMPS_40	4 (3.1)	162 (117)	0.282 (0.206)	0.011 (0.001)	4.0 (0.6)

a In parentheses: the same characteristics of 3D rGO–polymer aerogels synthesized by using the pretreatment step.^40^

b Nomenclature of the samples: reduction T_type of F.M. (NaSS or AMPS) copolymerized with MMA_weight % of the polymer (10 or 40).

**Figure 7:**
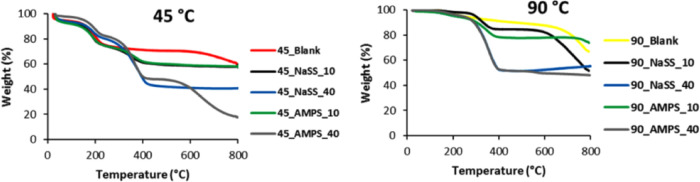
TGA curves of composite aerogels synthesized at 45 and 90 °C, with and without the pretreatment step at 80 °C.

According to the TGA thermographs shown in Figure 7, the composites present similar thermal degradation behavior as the blanks, except for one additional degradation step at about 300–400 °C assigned to the degradation of the polymers. This step is obviously higher for the composites containing 40% polymer than those with 10%. The fraction of oxygen functionalities in the composite aerogels is shown in Table 3 and refers only to the functional groups from rGO (hydroxyl, carboxyl, carbonyl, and epoxy) that are released in the temperature range of 100–250 °C, which is lower in the aerogels obtained at higher reduction temperatures. Compared to the composites produced with the pretreatment of GO dispersion (shown in brackets in Table 2),^40^ by avoiding this step, a much higher fraction of oxygen functionalities incorporated onto the graphenic surface of the aerogels was ensured. This difference is smaller in aerogels produced at a higher reduction temperature (90 °C).

**Figure 8:**
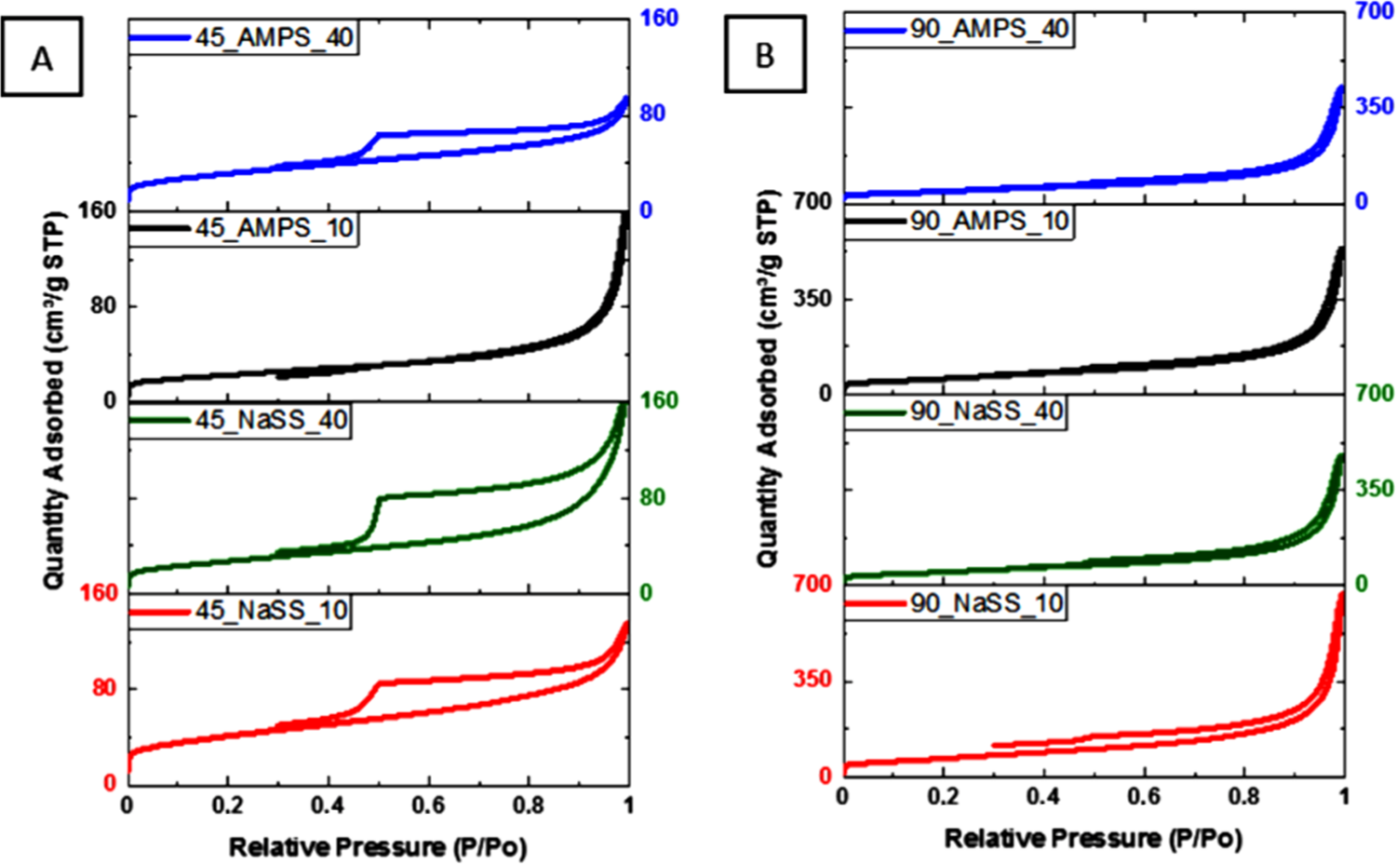
N_2_ adsorption–desorption isotherms of composite aerogels synthesized at 45 °C (A) and 90 °C (B) without pretreatment at 80 °C.

The N_2_ adsorption–desorption isotherms of the composite monoliths are shown in Figure 8. The textural properties of the composite monoliths are listed in Table 3. Figure 8 shows that the materials are mesoporous, but the hysteresis between the adsorption and desorption process is higher than that in the neat rGO aerogels, indicating further extension of different pore types within the composite aerogels. This effect is more pronounced when 40% polymer was added to the rGO aerogels, which confirms that the presence of the polymer affects the structure formation.

From Table 3, it can be seen that the BET surface area was importantly affected by the reduction temperature, giving rise to more compact and simultaneously more porous composites when produced at higher temperatures due to higher total pore volume. For example, the BET surface area increased from 147 m^2^/g in monolith 45_NaSS_10 to 157 m^2^/g in 60_NaSS_10 and to 246 m^2^/g in 90_NaSS_10. The aerogels synthesized at higher reduction temperatures are more densely packed and composed of a higher number of smaller pores. The pore volume increases with temperature from around 0.1 cm^3^/g in composites synthesized at 45 °C to 0.4 cm^3^/g at 90 °C. In terms of the volume of micropores and their contribution to the overall porous structure (% Micro), aerogels synthesized at 45 °C had much higher volumes of micropores than those produced at 90 °C.

The BET surface area, in most cases, decreases with the addition of particles in the structure compared to the blank materials (Table 2). During the synthesis, polymer particles were attached to the GO platelets, which altered their hydrophobicity and mobility, affecting the self-assembly and giving place to a lower total volume of pores. According to the previous experience, the addition of polymer particles in most of the cases increased the contribution of micropores (% Micro, Table 3) to the overall porous structure, as polymer particles act as spacers between the aggregated rGO layers, avoiding their total stacking.^40^ This effect was far weaker when the pretreatment step was skipped (Table 3), likely due to less initial aggregation of the GO platelets prior to the polymer addition and reduction, thus omitting the production of micro- and mesopores happening in this initial step of reduction.

**Figure 9:**
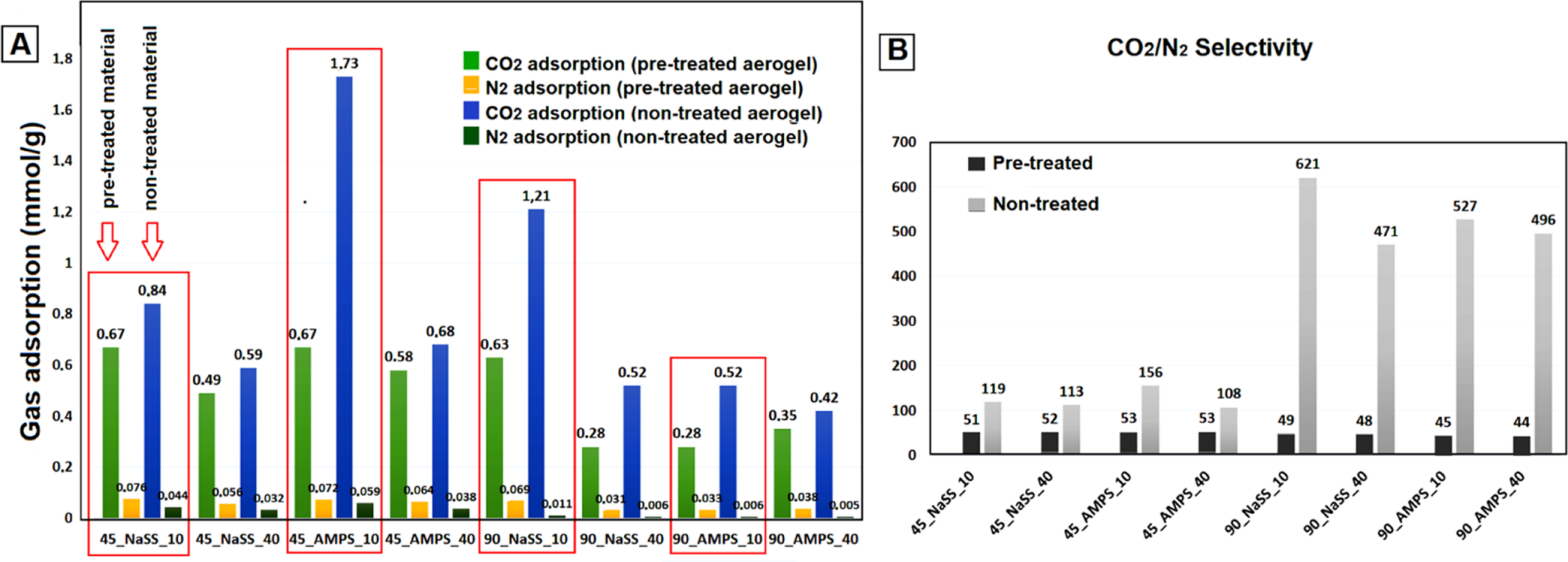
Comparison of CO_2_ and N_2_ adsorption (A). CO_2_/N_2_ selectivity (B) of composite aerogels functionalized with NaSS and AMPS.

The CO_2_ and N_2_ adsorption–desorption curves measured at 25 °C and up to 1 atm are presented in Figures S4 and S5, Supporting Information. The CO_2_ capturing performances of the aerogels at 25 °C and 1 atm are presented in Table S3 and in Figure 9A, where they are compared with the adsorption performance of aerogels produced with the pretreatment (composite monoliths prepared at 45 and 90 °C).^40^ In general, the addition of functionalized polymer particles did not improve the adsorption capacities compared to the blank materials, except in two cases of 45_AMPS_10 and 90_NaSS_10, for which higher CO_2_ adsorption values of 1.73 and 1.21 mmol/g, respectively, were achieved. N_2_ adsorption decreased with polymer addition and decreased significantly in the aerogels obtained at a high reduction temperature (90 °C). The pretreatment step results in more homogeneous characteristics with less difference between the different materials’ performance.

The CO_2_/N_2_ selectivity was calculated using the IAST method. The best fitting of the adsorption isotherms was obtained by the Freundlich model (eq 1). The fitting curves and the parameters of the Freundlich isotherm are shown in Figure S6 (for 45 °C) and Figure S7 (for 90 °C), and the results are presented in Table S3 and in Figure 9B. The addition of functionalized polymer particles in most of the aerogels did not significantly influence CO_2_ adsorption (with the exception of 45_AMPS_10 and 90_NaSS_10) but did affect the CO_2_/N_2_ selectivity. Regarding the polymer quantity, the addition of 10% polymer presented improved textural and adsorption properties in terms of CO_2_ uptake and higher selectivity than aerogels with a 40% polymer fraction. According to Figure 9A, the aerogels produced without pretreatment present increased CO_2_ adsorption compared to the pretreated ones, which probably is a consequence of an increased fraction of oxygen residual functionalities distributed over lower surface areas, resulting in denser functionalization. On the other hand, the N_2_ adsorption decreased for all of the nontreated aerogels without exception. These effects are more pronounced for aerogels produced at higher temperatures, resulting in a significant increase of the CO_2_/N_2_ selectivity (Figure 9B) to extraordinary values in a range of 471–621. The monoliths with the 40% polymer, when compared with monoliths with the 10%, generally present lower N_2_ adsorptions; nevertheless, the CO_2_ adsorption is also much lower, too. This effect has already been reported^40^ and was attributed to worsening textural properties when a higher polymer quantity was added due to the loss of micro and small mesopores.

According to Figure 9B, the highest selectivity value of 621 under the conditions studied was achieved by 90_NaSS_10, for which BET surface area was increased by avoiding the pretreatment, whereas the oxygen functionality fraction was lower. Besides that, the CO_2_ adsorption was duplicated from 0.63 to 1.21 mmol/g, and N_2_ adsorption dropped from 0.069 to 0.011 mmol/g, turning the material into greatly N_2_-phobic by avoiding the pretreatment step. Apparently, the interplay between both adsorption characteristics contributes to the best selectivity achieved, rising from 49 with the pretreatment to 621 when it was avoided. The selectivity was 13-fold increased, being between the highest values reported (Table 1). Taking into account that the behavior of these aerogels was opposite to that previously observed, it is clear that there are additional characteristics that affect the adsorption performance, which we will try to explain.

**Figure 10:**
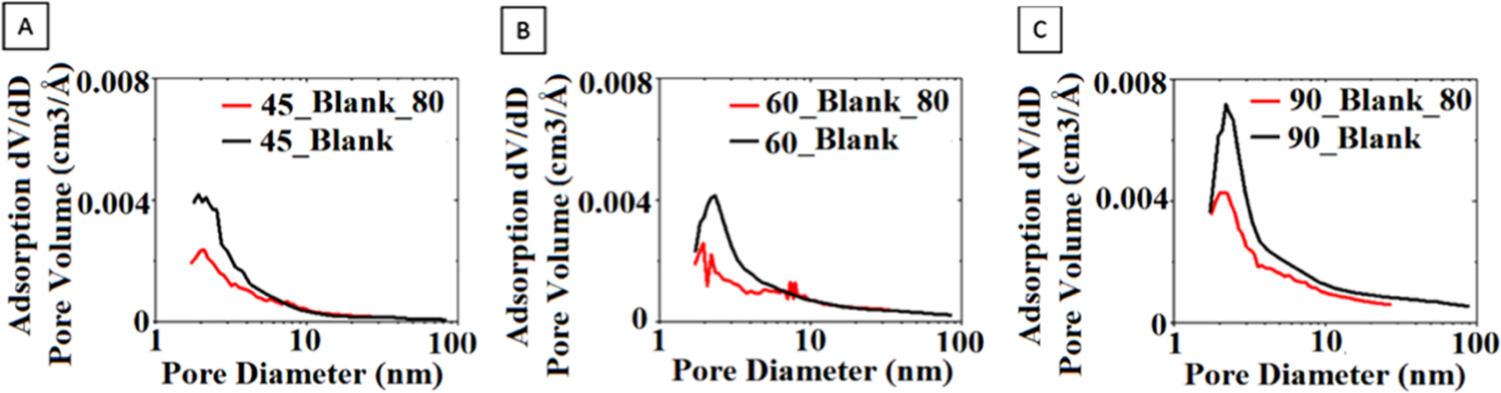
Pore size distribution of neat rGO aerogels at (A) 45 °C; (B) 60 °C; and (C) 90 °C, produced with and without pretreatment at 80 °C.

All aerogels produced at 90 °C without the pretreatment (neat and composites) presented more than 1 order of magnitude improved selectivity. A comparison of the characteristics of these monoliths, presented in Table 3, did not show important differences that might indicate the reason behind such a jump in the CO_2_/N_2_ selectivity. Theoretically, adsorbents with pore sizes between the kinetic diameter of CO_2_ (3.30 Å) and the one of N_2_ (3.64 Å) could introduce a molecular sieving effect and selectively adsorb CO_2_; nevertheless, such an effect is difficult to achieve experimentally because of the high similarity of both kinetic diameters. To check this, the pore size distributions of the aerogels were determined and compared. In Figure 10, the pore size distributions of the neat aerogels obtained with and without pretreatment at reduction temperatures of 45, 60, and 90 °C are presented. It can be observed that by avoiding the pretreatment, the fraction of the micro/mesopores in a range of 1–3 nm significantly increased with respect to the same fraction in pretreated aerogels, especially when the aerogel was produced at 90 °C.

**Figure 11:**
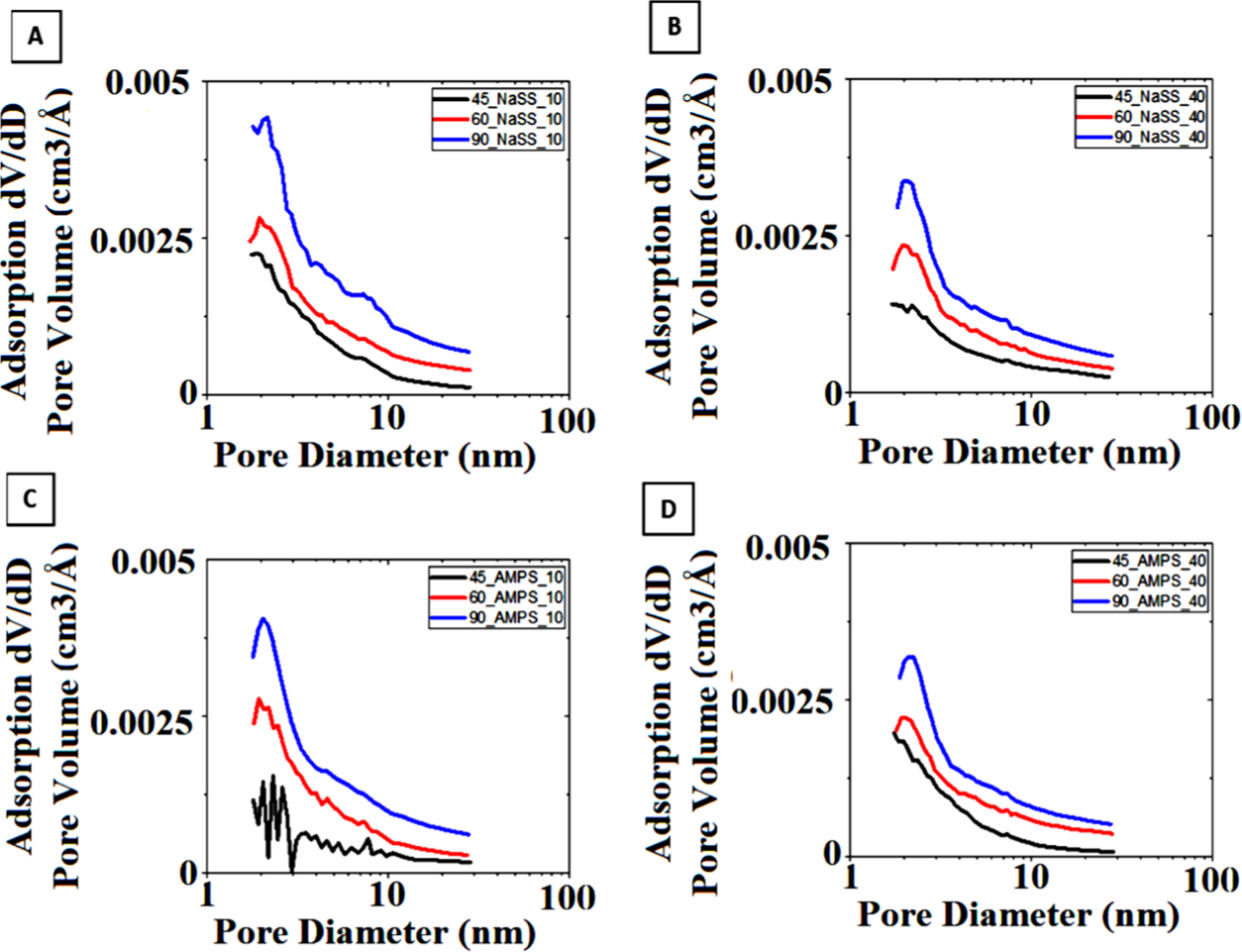
Pore size distribution of composite aerogels containing 10% NaSS-functionalized polymer (A), 40% NaSS-functionalized polymer (B), 10% AMPS-functionalized polymer (C), and 40% AMPS-functionalized polymer (D), synthesized without pretreatment.

Furthermore, the pore size distribution of the composites synthesized with the pretreatment step, recently published,^40^ and without the pretreatment from this work, shown in Figure 11, presents the same observation. The difference is pronounced for the aerogel produced at 90 °C containing NaSS-functionalized particles in 10% quantity, with respect to graphene. This aerogel (90_NaSS_10) is the one presenting the best improvement of CO_2_ capture and the highest selectivity of about 621, as already mentioned. This is in line with the reported observation acquired recently by applying deep learning to evaluate the CO_2_/N_2_ selectivity of porous carbon adsorbents for postcombustion carbon capture.^28,29^ Namely, they reported that bimodal pore size distributions with peaks at <2 nm and at about 5 nm (similar to that observed for 90_NaSS_10 in Figure 11) enhanced CO_2_/N_2_ selectivity because such mesopores disrupt and reduce N_2_ adsorption and 5 nm diameter mesopores critically favored CO_2_ adsorption. However, a higher fraction of pores in the mentioned region of 1–3 nm can be observed also when the reduction temperature is increased from 45 to 90 °C (Figure 10), which consequently increases the selectivity 4-fold for aerogels Blank_45 and Blank_90. It is clear that the same effect cannot be the reason behind the observed 13-fold enhanced selectivity of 90_NaSS_10. Moreover, as Figure 11 shows, the rest of the composite aerogels present a continuous pore distribution (not bimodal), containing a larger fraction of micropores along with small mesopores up to 5 nm, which according to the reported deep learning prediction, gave rise to lower selectivities.^29^ The composite aerogels from our work present high selectivity, indicating the presence of additional influencing parameters. In order to shed a bit of light on this issue, deeper characterization of the surface of the aerogels was performed.

In order to go further in the N_2_ adsorption understanding, the surface chemistry of the monoliths focused on blank materials was profoundly studied using XPS. Namely, by TGA, the whole oxidized quantity of the materials was determined, and probably some of the functional groups might be buried within the structures, in which case, they would not contribute to the adsorption process. In Figure S8 in the Supporting Information, the survey scan spectra of all samples are presented, in which mostly the presence of carbon and oxygen are identified. Figure S9 shows the high-resolution XPS spectra of the C 1s region for different monoliths, in which the deconvoluted peaks are assigned to the chemical moieties according to the binding energy of the peaks. It is clear that all of the monoliths made of neat rGO present the same peaks at 284.6, 287.0, and 288.6 eV, whose binding energies correspond to C–C, C–O, and C=O or O–C=O, respectively. Even though their fraction is affected by the reduction temperature and by the procedure of synthesis (with or without the pretreatment step), the differences are rather negligible, as shown in Table 4.

**Table 4:** XPS Data for 45_Blank_80, 45_Blank, 60_Blank_80, 60_Blank, 90_Blank_80, and 90_Blank

sample	cycle	groups	binding energy (eV)	Conc. %	Conc. %
45_Blank_80	C	C–C, C–H	284.6	39.9	74.0
		C–O	287.0	25.5	
		C=O, O–C=O	288.6	5.7	
		π–π* sat.	290.8	2.5	
		π–π* sat.	293.9	0.3	
	O	O–C=O, C=O	531.2	3.2	23.5
		C–OH	532.8	19.3	
		C–O–C	535.0	0.9	
45_Blank	C	C–C, C–H	284.6	39.1	74.6
		C–O	286.9	24.1	
		C=O, O–C=O	288.4	7.6	
		π–π* sat.	290.8	3.1	
		π–π* sat.	293.8	0.7	
	O	O–C=O, C=O	531.2	2.1	24.7
		C–OH	532.9	21.7	
		C–O–C	535.1	0.9	
60_Blank_80	C	C–C, C–H	284.6	44.9	80.4
		C–O	286.7	21.7	
		C=O, O–C=O	288.7	6.3	
		π–π* sat.	290.9	6.0	
		π–π* sat.	294.0	1.6	
	O	O–C=O, C=O	531.2	2.5	17.9
		C–OH	532.8	14.5	
		C–O–C	535.6	0.9	
60_Blank	C	C–C, C–H	284.6	47.3	83.1
		C–O	286.7	20.4	
		C=O, O–C=O	288.6	7.1	
		π–π* sat.	290.9	6.4	
		π–π* sat.	293.9	1.9	
	O	O–C=O, C=O	531.3	3.3	15.2
		C–OH	532.9	11.0	
		C–O–C	535.3	0.9	
90_Blank_80	C	C–C, C–H	284.6	50.1	87.3
		C–O	286.1	18.7	
		C=O, O–C=O	288.4	9.1	
		π–π* sat.	291.1	7.6	
		π–π* sat.	294.1	1.7	
	O	O–C=O, C=O	531.5	3.9	11.7
		C–OH	533.2	6.8	
		C–O–C	535.6	1.0	
90_Blank	C	C–C, C–H	284.6	44.2	82.6
		C–O	286.2	20.4	
		C=O, O–C=O	288.3	9.1	
		π–π* sat.	290.8	7.1	
		π–π* sat.	293.9	1.7	
	O	O–C=O, C=O	531.4	4.8	15.8
		C–OH	533.1	9.7	
		C–O–C	535.4	1.4	

Table 4 presents the exact position of the peaks and the corresponding binding energies, as well as the assignment of the peaks and the relative fraction of each of the functional groups within the respective aerogel. The trace quantities of N and Si observed in the samples are not shown in Table 4. The concentrations of both C and O show that in all cases, the aerogels produced by avoiding the pretreatment have more oxidized surfaces; thus, they are richer in oxygen functional groups. Moreover, the XPS spectra show that there are π–π interactions established between the individual graphene sheets, the quantity of which increased in the structures produced at higher temperatures.

Table 4 demonstrates that the reduction temperature certainly affects the fraction of oxygen-containing moieties on the surface. By increasing the reduction temperature from 45 to 90 °C, the quantity of residual surface functional groups in the aerogels decreases; i.e., there is greater recuperation of the sp^2^-hybridized carbons in the aerogels synthesized at higher temperatures. The pretreated GO resulted in less functionalization than the aerogels produced from non-pretreated GO, except at 60 °C, for which the effect is just the opposite. However, all these aerogels presented improved selectivity when the pretreatment was skipped (Figure 4), indicating that the selectivity enhancement is not related to the increased oxygen functionalization. The aerogels produced at 45 °C with and without pretreatment present only a 1% difference in the content of surface oxygen functionalities, and the selectivity increases from 96 to 106 (Figure 4) by avoiding the pretreatment. The ones produced at 90 °C present a difference of about 4% in surface functionality content, but the selectivity grows from 86 to 470, so the higher functionalization cannot justify the selectivity rise.

Recent theoretical studies have shown that nitrogen molecules prefer adsorption onto graphenic surfaces that contain defects and vacancies.^50^ Taking into account that graphene-based materials, when produced at higher temperatures, are less defected due to more efficient recuperation of the sp^2^-hybridized carbon structure, this may explain the observed lower N_2_ adsorption by the aerogels produced at 90 °C. Nevertheless, the huge difference observed between both materials synthesized at 90 °C (with and without pretreatment) remains inexplicable. Considering that the pretreatment at 80 °C would produce structural readjustments and correction of the vacancies,^51^ one would expect that the treated materials should have lower N_2_ adsorption, which is opposite to that observed.

The rGO surface morphology can be as well affected by the pretreatment of GO at 80 °C, altering subsequently the interaction of the aerogels with CO_2_ and N_2_ molecules. To obtain more details on the surface morphology of the aerogels or, in other words, to check how the pretreatment affects the morphology of the platelets that build the 3D aerogel structure, AFM was used to survey the surface. Therefore, it was attempted to synthesize the same structures but avoid the creation of 3D monolithic morphology by addition of intense agitation during the GO reduction. The reduction was performed at 45 and 90 °C. It is worth mentioning that due to the high temperature, the samples at 90 °C created monolithic structures, so they were not studied further. Oppositely, at 45 °C, a powder material was produced, and the difference between the pretreated and non-pretreated ones was studied by AFM.

**Figure 12:**
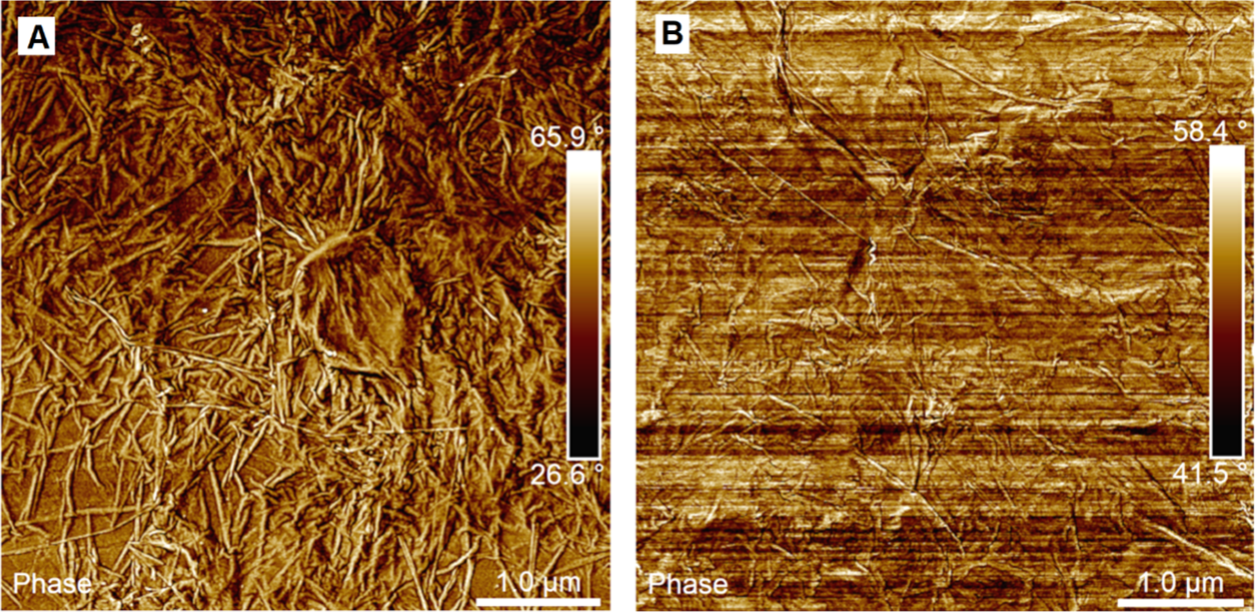
AFM images for pretreated rGO (A) and nontreated rGO (B) produced by reduction at 45 °C under rigorous agitation.

Figure 12A,B presents the AFM phase images of the rGO material obtained by reduction at 45 °C of treated and nontreated GO, respectively, offering qualitative evaluation of the surface properties. Both materials present similar phases, likely corresponding to the oxidized graphenic surface, but irregular and rough. The treated rGO (Figure 12A) is much more wrinkled, likely because it is less oxidized and, thus, more elastic. Consequently, numerous wrinkles forming curved surfaces with a diameter of about 100 nm can be observed. Oppositely, the nontreated rGO (Figure 12B) presents a morphology of crumpled paper, with fewer wrinkles, that moreover seems to have sharper edges. Considering that the nontreated material is more oxidized, as demonstrated by TGA and XPS results, it is simultaneously stiffer, which explains the surface morphology observed.

A recent quantum mechanical study has demonstrated higher potentials for N_2_–C on more elastic and curved carbon structures (such as fullerenes or C168 schwarzite) than in graphite, resulting in increased adsorption energies for N_2_ on these surfaces.^52^ Therefore, the highly wrinkled surface observed in the pretreated rGO can be the final and probably the critical parameter that determines their higher N_2_ adsorption. On the other hand, the not-treated rGO stiffer surfaces, which moreover formed a higher fraction of micro- and small mesopores, present a low adsorption potential of N_2_ adsorption, forming an even almost completely N_2_-phobic material when created at 90 °C. It is worth mentioning that even though 90 °C-reduced rGO platelet morphology could not be studied by AFM, it is for expectation that these differences would be even more pronounced, explaining the improved selectivity for the aerogels produced at 90 °C.

## Conclusions

In this study, porous monolithic aerogel adsorbents for CO_2_ were prepared, based on 3D graphene and graphene–polymer structures, for potential use in CCS postcombustion technology. The monolithic materials were synthesized by combined chemical and thermal reduction of single-layer GO platelets in aqueous dispersions, performed at 45, 60, and 90 °C. During this process, the rGO and rGO–polymer flakes were self-assembled, building the 3D porous aerogels. The main aim of this work was to increase the CO_2_/N_2_ selectivity, as an important parameter that significantly affects the costs of the CCS process. The selectivity elevation was projected through the increase of the fraction of the oxygen functional groups and that of the small mesopores within the rGO-based monoliths by manipulation of the synthesis procedure.

Certainly, the modified synthesis procedure induced a rise in the fraction of oxygen functionalities and an increase in the microporosity, resulting in higher CO_2_ and lower N_2_ adsorption capacities in most of the aerogels. This effect was huge for the monoliths produced at the highest temperature (90 °C), resulting in a rise of CO_2_/N_2_ selectivity to values of 470–621, which is more than 1 order of magnitude higher than the reference monoliths. Moreover, the selectivity values achieved in this work are far higher than all of the reported values for carbon-based nanomaterial adsorbents to the best of the authors’ knowledge.

The attempts to obtain a deeper understanding of the issue have revealed that by the modified synthesis procedure, the aerogels produced are oxidized more on the surface, they have a higher contribution of micropores and small mesopores to the surface area, and the surface morphology of the graphene platelets was also impacted. Namely, stiffer and more rigid surfaces were produced, which likely induced less N_2_ adsorption. On the other hand, the densely functionalized surface of these aerogels likely offered more adsorption sites to CO_2_ molecules, resulting in the outstanding selectivity attained.

To the best of the authors’ knowledge, this is the first experimental study that associates the elasticity and curvature of the graphene surface (or carbon-based nanomaterials in general) with the N_2_-phobicity and selectivity of CO_2_ adsorbents. It offers new viewpoints for the improvement of the selectivity of C-based nanomaterials adsorbents for application in postcombustion CCS.

## Supporting Information Available

Formulation employed for the synthesis of both NASS-MMA and AMPS-MMA latexes; chemical structures of NaSS and AMPS functional monomers; details on the synthesis of composite monoliths; CO_2_ and N_2_ adsorption capacity at 25 °C and 1 atm and IAST selectivity for neat rGO aerogels; CO_2_ adsorption–desorption and N_2_ adsorption isotherms of neat rGO aerogels; Freundlich equation fitting curves for the CO_2_ and N_2_ isotherms for neat rGO aerogels; CO_2_ adsorption–desorption and N_2_ adsorption isotherms for composite aerogels containing 10 and 40% polymer synthesized at 45 and 90 °C without pretreatment; CO_2_ and N_2_ adsorption capacity and selectivity for composite aerogels; in brackets, the results of the previously reported 3D rGO–polymer structures synthesized by using the pretreatment step are also presented; Freundlich equation fitting curve for CO_2_ and N_2_ isotherms for composite aerogels synthesized at 45 and 90 °C without pretreatment; XPS survey spectra of 45_Blank_80, 45_Blank, 60_Blank_80, 60_Blank, 90_Blank_80, and 90_Blank aerogels; and high-resolution C 1s XPS spectra of (A) 45_Blank_80, (B) 45_Blank, (C) 60_Blank_80, (D) 60_Blank, (E) 90_Blank_80, and (F) 90_Blank aerogels (PDF)

## References

[ref1] Höök M.; Tang X. Depletion of fossil fuels and anthropogenic climate change-A review. Energy Policy 2013, 52, 797–809. 10.1016/j.enpol.2012.10.046.

[ref2] Anderson T. R.; Hawkins E.; Jones P. D. CO2, the greenhouse effect and global warming: from the pioneering work of Arrhenius and Callendar to today’s Earth System Models. Endeavour 2016, 40 (3), 178–187. 10.1016/j.endeavour.2016.07.002. 27469427

[ref3] Salvi B. L.; Jindal S. Recent developments and challenges ahead in carbon capture and sequestration technologies. SN Appl. Sci. 2019, 1 (8), 885 10.1007/s42452-019-0909-2.

[ref4] Leung D. Y. C.; Caramanna G.; Maroto-Valer M. M. An overview of current status of carbon dioxide capture and storage technologies. Renewable Sustainable Energy Rev. 2014, 39, 426–443. 10.1016/j.rser.2014.07.093.

[ref5] Ben-Mansour R.; Habib M. A.; Bamidele O. E.; Basha M.; Qasem N. A. A.; Peedikakkal A.; Laoui T.; Ali M. Carbon capture by physical adsorption: Materials, experimental investigations and numerical modeling and simulations - A review. Appl. Energy 2016, 161, 225–255. 10.1016/j.apenergy.2015.10.011.

[ref6] Dutcher B.; Fan M.; Russell A. G. Amine-based CO2 capture technology development from the beginning of 2013-A review. ACS Appl. Mater. Interfaces 2015, 7 (4), 2137–2148. 10.1021/am507465f. 25607244

[ref7] Luis P. Use of monoethanolamine (MEA) for CO2 capture in a global scenario: Consequences and alternatives. Desalination 2016, 380, 93–99. 10.1016/j.desal.2015.08.004.

[ref8] Chao C.; Deng Y.; Dewil R.; Baeyens J.; Fan X. Post-combustion carbon capture. Renewable Sustainable Energy Rev. 2021, 138, 110490 10.1016/j.rser.2020.110490.

[ref9] Raganati F.; Miccio F.; Ammendola P. Adsorption of Carbon Dioxide for Post-combustion Capture: A Review. Energy Fuels 2021, 35 (16), 12845–12868. 10.1021/acs.energyfuels.1c01618.

[ref10] Choi S.; Drese J. H.; Jones C. W. Adsorbent materials for carbon dioxide capture from large anthropogenic point sources. ChemSusChem 2009, 2 (9), 796–854. 10.1002/cssc.200900036. 19731282

[ref11] Ahmed R.; Liu G.; Yousaf B.; Abbas Q.; Ullah H.; Ali M. U. Recent advances in carbon-based renewable adsorbent for selective carbon dioxide capture and separation-A review. J. Cleaner Prod. 2020, 242, 118409 10.1016/j.jclepro.2019.118409.

[ref12] Park J.; Cho S. Y.; Jung M.; et al. Efficient synthetic approach for nanoporous adsorbents capable of pre-and post-combustion CO2capture and selective gas separation. J. CO2 Util. 2021, 45, 101404 10.1016/j.jcou.2020.101404.

[ref13] Qiao Z.; Wang Z.; Zhang C.; Yuan S.; Zhu Y.; Wang J.; Wang S. PVAm–PIP/PS composite membrane with high performance for CO2/N2 separation. AIChE J. 2013, 59 (4), 215–228. 10.1002/aic.13781.

[ref14] Oschatz M.; Antonietti M. A search for selectivity to enable CO2 capture with porous adsorbents. Energy Environ. Sci. 2018, 11 (1), 57–70. 10.1039/C7EE02110K.

[ref15] Zhao Y.; Liu X.; Han Y. Microporous carbonaceous adsorbents for CO2 separation via selective adsorption. RSC Adv. 2015, 5 (38), 30310–30330. 10.1039/C5RA00569H.

[ref16] Belmabkhout Y.; Guillerm V.; Eddaoudi M. Low concentration CO2 capture using physical adsorbents: Are metal-organic frameworks becoming the new benchmark materials?. Chem. Eng. J. 2016, 296, 386–397. 10.1016/j.cej.2016.03.124.

[ref17] Ho M. T.; Allinson G. W.; Wiley D. E. Reducing the cost of CO2 capture from flue gases using pressure swing adsorption. Ind. Eng. Chem. Res. 2008, 47 (14), 4883–4890. 10.1021/ie070831e.

[ref18] Ma X.; Su C.; Liu B.; Wu Q.; Zhou K.; Zeng Z.; Li L. Heteroatom-doped porous carbons exhibit superior CO2 capture and CO2/N2 selectivity: Understanding the contribution of functional groups and pore structure. Sep. Purif. Technol. 2021, 259, 118065 10.1016/j.seppur.2020.118065.

[ref19] Liang W.; Liu Z.; Peng J.; Zhou X.; Wang X.; Li Z. Enhanced CO 2 Adsorption and CO 2 /N 2 /CH 4 Selectivity of Novel Carbon Composites CPDA@A-Cs. Energy Fuels 2019, 33 (1), 493–502. 10.1021/acs.energyfuels.8b03637.

[ref20] Alabadi A. A.; Abbood H. A.; Dawood A. S.; Tan B. Ultrahigh-CO2 Adsorption Capacity and CO2/N2 Selectivity by Nitrogen-Doped Porous Activated Carbon Monolith. Bull. Chem. Soc. Jpn. 2020, 93 (3), 421–426. 10.1246/bcsj.20190336.

[ref21] Wu Y.; Wang J.; Muhammad Y.; Subhan S.; Zhang Y.; Ling Y.; Li J.; Zhao Z.; Zhao Z. Pyrrolic N-enriched carbon fabricated from dopamine-melamine via fast mechanochemical copolymerization for highly selective separation of CO2 from CO2/N2. Chem. Eng. J. 2018, 349, 92–100. 10.1016/j.cej.2018.05.072.

[ref22] Ning H.; Yang Z.; Wang D.; Meng Z.; Li Y.; Ju X.; Wang C. Graphene-based semi-coke porous carbon with N-rich hierarchical sandwich-like structure for efficient separation of CO2/N2. Microporous Mesoporous Mater. 2021, 311, 110700 10.1016/j.micromeso.2020.110700.

[ref23] Li Q.; Liu S.; Wang L.; Chen F.; Shao J.; Hu X. Efficient nitrogen doped porous carbonaceous CO2 adsorbents based on lotus leaf. J. Environ. Sci. 2021, 103, 268–278. 10.1016/j.jes.2020.11.008. 33743908

[ref24] Yue L.; Rao L.; Wang L.; An L.; Hou C.; Ma C.; DaCosta H.; Hu X. Efficient CO2 Adsorption on Nitrogen-Doped Porous Carbons Derived from d -Glucose. Energy Fuels 2018, 32 (6), 6955–6963. 10.1021/acs.energyfuels.8b01028.

[ref25] An L.; Liu S.; Wang L.; Wu J.; Wu Z.; Ma C.; Yu Q.; Hu X. Novel Nitrogen-Doped Porous Carbons Derived from Graphene for Effective CO 2 Capture. Ind. Eng. Chem. Res. 2019, 58 (8), 3349–3358. 10.1021/acs.iecr.8b06122.

[ref26] Buß F.; Mehlmann P.; Mück-Lichtenfeld C.; Bergander K.; Dielmann F. Reversible Carbon Dioxide Binding by Simple Lewis Base Adducts with Electron-Rich Phosphines. J. Am. Chem. Soc. 2016, 138 (6), 1840–1843. 10.1021/jacs.5b13116. 26824487

[ref27] Liu Y.; Wilcox J. Effects of surface heterogeneity on the adsorption of CO 2 in microporous carbons. Environ. Sci. Technol. 2012, 46 (3), 1940–1947. 10.1021/es204071g. 22216997

[ref28] Wang S.; Zhang Z.; Dai S.; Jiang D. E. Insights into CO2/N2 Selectivity in Porous Carbons from Deep Learning. ACS Mater. Lett. 2019, 1 (5), 558–563. 10.1021/acsmaterialslett.9b00374.

[ref29] Wang S.; Li Y.; Dai S.; Jiang D. Prediction by Convolutional Neural Networks of CO2/N2 Selectivity in Porous Carbons from N2 Adsorption Isotherm at 77 K. Angew. Chem., Int. Ed. 2020, 59 (44), 19645–19648. 10.1002/anie.202005931. 32485029

[ref30] Lee J. H.; Lee H. J.; Lim S. Y.; Kim B. G.; Choi J. W. Combined CO2-philicity and Ordered Mesoporosity for Highly Selective CO2 Capture at High Temperatures. J. Am. Chem. Soc. 2015, 137 (22), 7210–7216. 10.1021/jacs.5b03579. 26000786

[ref31] Patel H. A.; Je S. H.; Park J.; Jung Y.; Coskun A.; Yavuz C. T. Directing the structural features of N2-phobic nanoporous covalent organic polymers for CO2 capture and separation. Chem. - Eur. J. 2014, 20 (3), 772–780. 10.1002/chem.201303493. 24338860

[ref32] Sui Z. Y.; Han B. H. Effect of surface chemistry and textural properties on carbon dioxide uptake in hydrothermally reduced graphene oxide. Carbon 2015, 82, 590–598. 10.1016/j.carbon.2014.11.014.

[ref33] Liu F. Q.; Wang L. L.; Li G. H.; Li W.; Li C. Q. Hierarchically structured graphene coupled microporous organic polymers for superior CO2 capture. ACS Appl. Mater. Interfaces 2017, 9 (39), 33997–34004. 10.1021/acsami.7b11492. 28905620

[ref34] Kwac K.; Lee J. H.; Choi J. W.; Jung Y. Computational Analysis of Pressure-Dependent Optimal Pore Size for CO2 Capture with Graphitic Surfaces. J. Phys. Chem. C 2016, 120 (7), 3978–3985. 10.1021/acs.jpcc.5b12404.

[ref35] Lee J. H.; Kwac K.; Lee H. J.; Lim S. Y.; Jung D. S.; Jung Y.; Choi J. W. Optimal Activation of Porous Carbon for High Performance CO2 Capture. ChemNanoMat 2016, 2 (6), 528–533. 10.1002/cnma.201600082.

[ref36] Lee J. H.; Lee H. J.; Choi J. W. Unveiling anomalous CO2-to-N2 selectivity of graphene oxide. Phys. Chem. Chem. Phys. 2017, 19 (34), 22743–22748. 10.1039/C7CP04318J. 28825756

[ref37] Saha D.; Kienbaum M. J. Role of oxygen, nitrogen and sulfur functionalities on the surface of nanoporous carbons in CO2 adsorption: A critical review. Microporous Mesoporous Mater. 2019, 287, 29–55. 10.1016/j.micromeso.2019.05.051.

[ref38] Politakos N.; Barbarin I.; Cantador L. S.; Cecilia J. A.; Mehravar E.; Tomovska R. Graphene-Based Monolithic Nanostructures for CO2 Capture. Ind. Eng. Chem. Res. 2020, 59 (18), 8612–8621. 10.1021/acs.iecr.9b06998.

[ref39] Politakos N.; Barbarin I.; Cordero-Lanzac T.; Gonzalez A.; Zangi R.; Tomovska R. Reduced graphene oxide/polymer monolithic materials for selective CO2 capture. Polymers 2020, 12 (4), 936 10.3390/polym12040936. 32316554 PMC7240369

[ref40] Barbarin I.; Politakos N.; Serrano-Cantador L.; Cecilia J. A.; Sanz O.; Tomovska R. Towards functionalized graphene/polymer monolithic structures for selective CO2 capture. Microporous Mesoporous Mater. 2022, 337, 111907 10.1016/j.micromeso.2022.111907.

[ref41] Pan Y.; Xue M.; Chen M.; Lim S. Y.; Jung D. S.; Jung Y.; Choi J. W. ZIF-derived: In situ nitrogen decorated porous carbons for CO2 capture. Inorg. Chem. Front. 2016, 3 (9), 1112–1118. 10.1039/C6QI00158K.

[ref42] Chandra V.; Yu S.; Kim S. H.; Yoon Y. S.; Kim D. Y.; Kwon A. H.; Meyyappan M.; Kim K. S. Highly selective CO2 capture on N-doped carbon produced by chemical activation of polypyrrole functionalized graphene sheets. Chem. Commun. 2012, 48 (5), 735–737. 10.1039/C1CC15599G. 22117227

[ref43] Chowdhury S.; Balasubramanian R. Highly efficient, rapid and selective CO2 capture by thermally treated graphene nanosheets. J. CO2 Util. 2016, 13, 50–60. 10.1016/j.jcou.2015.12.001.

[ref44] Chowdhury S.; Balasubramanian R. Holey graphene frameworks for highly selective post-combustion carbon capture. Sci. Rep. 2016, 6, 21537 10.1038/srep21537. 26879393 PMC4754909

[ref45] Chowdhury S.; Balasubramanian R. Three-Dimensional Graphene-Based Porous Adsorbents for Postcombustion CO2 Capture. Ind. Eng. Chem. Res. 2016, 55 (29), 7906–7916. 10.1021/acs.iecr.5b04052.

[ref46] Myers A. L.; Prausnitz J. Thermodynamics of Mixed-Gas Adsorption. AIChE J. 1965, 11 (1), 121–126. 10.1002/aic.690110125.

[ref47] Webb P. A.; Orr C. Analytical Methods in Fine Particle Technology; Micromeritics Instrument Corporation, 1997; pp 54–58.

[ref48] Brandrup J.; Immergnt E. H. Polymer Handbook, 3rd ed.; Wiley: New York, 1989.

[ref49] Meconi G. M.; Tomovska R.; Zangi R. Adsorption of CO 2 gas on graphene-polymer composites. J. CO2 Util. 2019, 32, 92–105. 10.1016/j.jcou.2019.03.005.

[ref50] Vallejo E.; López-Pérez P. A. Strong chemical adsorption of CO2 and N2 on a five-vacancy graphene surface. Solid State Commun. 2022, 356, 114934 10.1016/j.ssc.2022.114934.

[ref51] Athanasekou C. P.; Pedrosa M. F.; Silva A. M. T.; Psycharis V. P.; Romanos G. E. Mild temperature-gas separation performance of graphene oxide membranes for extended period: micropore to meso- and macropore readjustments and the fate of membranes under the influence of dynamic graphene oxide changes. Chem. Eng. J. Adv. 2021, 5, 100066 10.1016/j.ceja.2020.100066.

[ref52] Klauda J. B.; Jiang J.; Sandler S. I. An ab initio study on the effect of carbon surface curvature and ring structure on N2(O2) - Carbon intermodular potentials. J. Phys. Chem. B 2004, 108 (28), 9842–9851. 10.1021/jp037897h.

