# Supplementary material for: Resembling Graphene/Polymer Aerogel Morphology for Advancing the CO2/N2 Selectivity of the Postcombustion CO2 Capture Process
Source: Ind Eng Chem Res. 2024 Apr 9;63(16):7073–87. doi: 10.1021/acs.iecr.3c02989 (PMC11048490; doi:10.1021/acs.iecr.3c02989)
Supplement: Supplementary file 1 — ie3c02989_si_001.pdf [file ie3c02989_si_001.pdf]

Supporting Information

Resembling graphene/polymer aerogels morphology for advancing the CO_2/N_2 selectivity of post-combustion CO_2 capture process

Iranzu Barbarin,[a] Monika Fidanchevska,[a] Nikolaos Politakos,[a] Luis Serrano-Cantador,[b] Juan Antonio Cecilia,[c] Dolores Martín,[d] Oihane Sanz,[e] and Radmila Tomovska,[a,f,*]

[a]POLYMAT and Department of Applied Chemistry, University of the Basque Country UPV/EHU, 20018 Donostia-San Sebastián, Spain.

[b]Biopren Group, Inorganic Chemistry and Chemical Engineering Department, Nanochemistry University Institute (IUNAN), Universidad de Córdoba, 14014 Córdoba, Spain.

[c]Inorganic Chemistry, Crystallography and Mineralogy, University of Málaga, 29071 Málaga, Spain

[d]Macrobehaviour-Mesostructure-Nanotechnology SGIker Service, Faculty of Engineering of Gipuzkoa, University of the Basque Country (UPV/EHU), Plaza Europa 1, 20018, Donostia-San Sebastian, Spain

[e]Department of Applied Chemistry, University of the Basque Country, 20018 Donostia-San Sebastián, Spain.

[f]Ikerbasque, Basque Foundation for Science, Maria Diaz de Haro 3, 48013, Bilbao, Spain

*Corresponding author: radmila.tomovska@ehu.eus

Table S1. Formulation employed for the synthesis of both NASS-MMA and AMPS-MMA latexes. F.M. (functional monomer) corresponds to either NaSS or AMPS.

		Initial seed (g)	MMA (g)	F.M. (g)	Dowfax (g)	KPS (g)	AsA (g)	TBHP (g)	H_2O (g)
**Batch**	Seed		360		7.2	1.8			1440
**Semibatch**	Initial charge	33.87	-	-	-	-	-	-	120
	F1	-	-	1.05	-	-	0.53	-	57.85
	F2	-	98.28	-	1.05	-	-	0.53	40

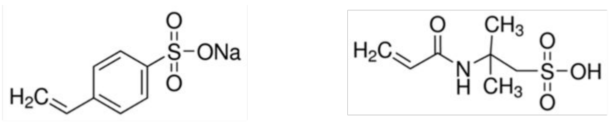

Sodium 4-vinylbenzensulfonate (NaSS)

2-Acrylamido-2-methyl-1-propanesulfonic acid (AMPS)

Figure S1. Chemical structures of NaSS and AMPS functional monomers.

Details on the synthesis of composite monoliths

The composite aerogels were synthesized by mixing of polymer particle dispersion (latex) with GO dispersions in appropriate ratio to give rise to 10% and 40% polymer fraction by weight within the composite materials. Afterwards, ascorbic acid reducing agent was added and the mixture was subjected to increased temperature to reduce the GO platelets and to produce the monoliths.

When the dispersions were mixed for 30 min prior to reduction process, it was observed that black monolith hydrogel was obtained. However, instead of clean water around the monolith, it was still milky white, indicating presence of polymer particles in the residual water (the latex is milky white naturally). This has shown to us that all rGO was incorporated into the structure, but only part of the polymer particles from the latex.

To improve the polymer incorporation into the monoliths, the mixing time of GO and polymer particles was prolonged up to 3h prior to the reduction process. It was found that after 2h mixing; the residual water in which the black monolith was floating was completely transparent, indicating no presence of polymer particles. The residual water was analyzed gravimetrically to confirm it. We dried it and measured the solids remaining, finding very small quantities (negligible), and probably coming from some reduction side products.

This indicated that at larger mixing time of both dispersions, the small polymer particles adsorbed completely onto rGO. During reduction process, these composite platelets self-assembled forming the monolithic hydrogel, that after freeze-drying procedure was turned into composite aerogels.

Table S2. CO_2 and N_2 adsorption capacity at 25ºC and 1 atm and IAST selectivity for neat rGO aerogels.

Material	CO_2 adsorption (mmol g^{-1})	N_2 adsorption (mmol g^{-1})	Selectivity
**45_Blank_80**	1.01	0.064	90
**45_Blank**	1.29	0.073	106
**60_Blank_80**	0.95	0.088	62
**60_Blank**	1.02	0.053	85
**90_Blank_80**	0.94	0.061	86
**90_Blank**	0.67	0.008	470

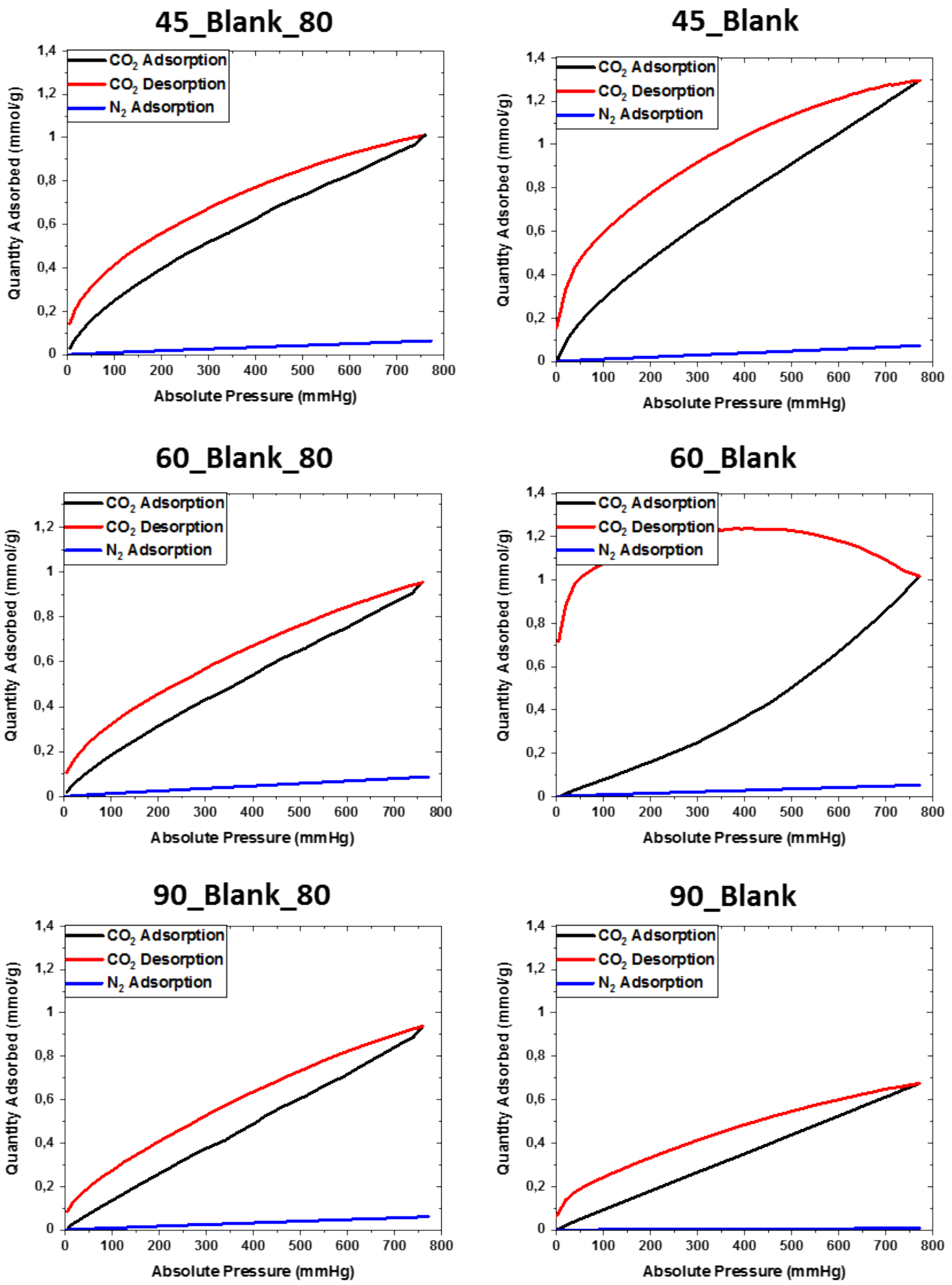

Figure S2. CO_2 adsorption-desorption and N_2 adsorption isotherms of neat rGO aerogels

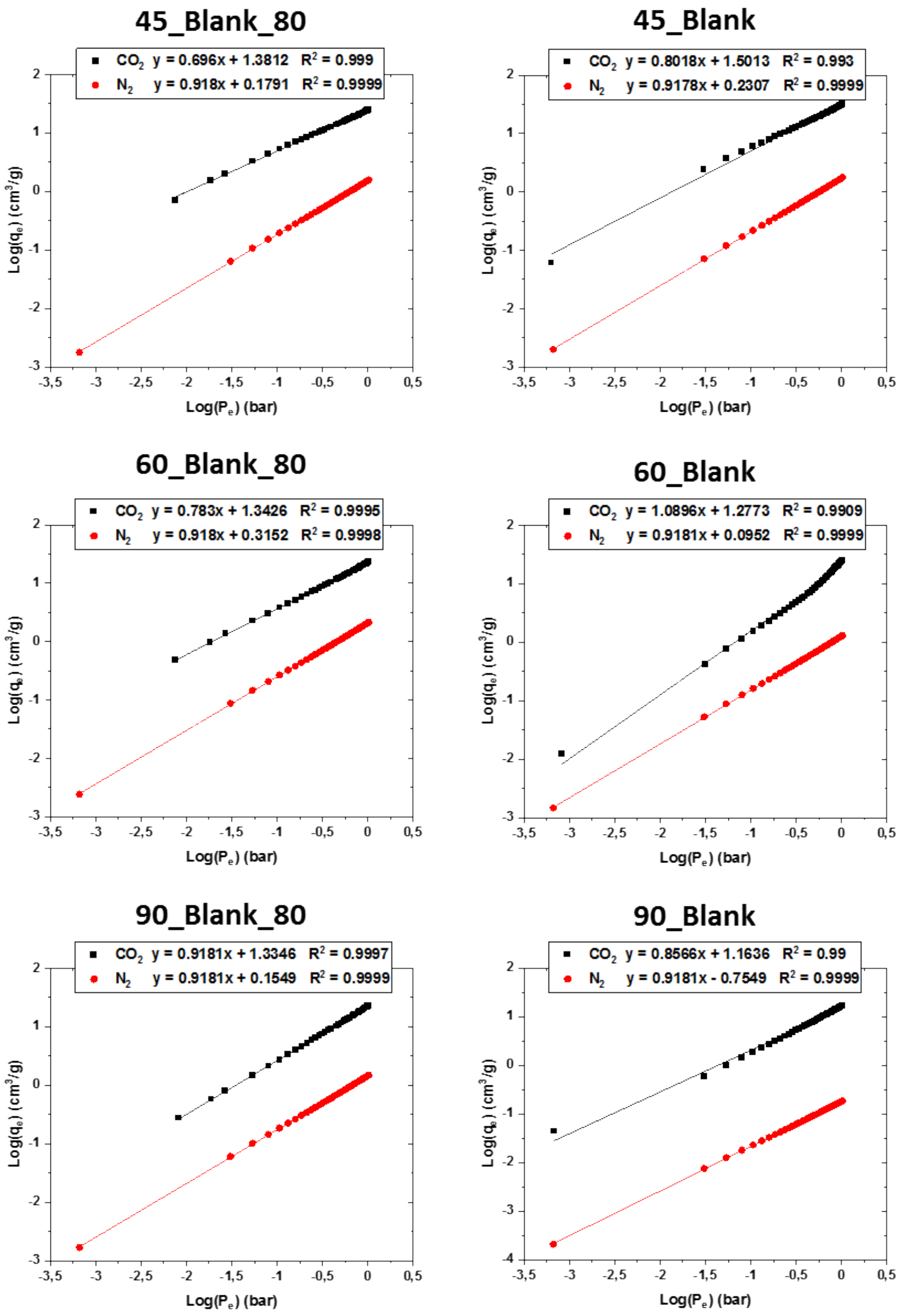

Figure S3. Freundlich equation fitting curves for CO_2 isotherm (black curve) and for N_2 isotherm (red curve) for neat rGO aerogels.

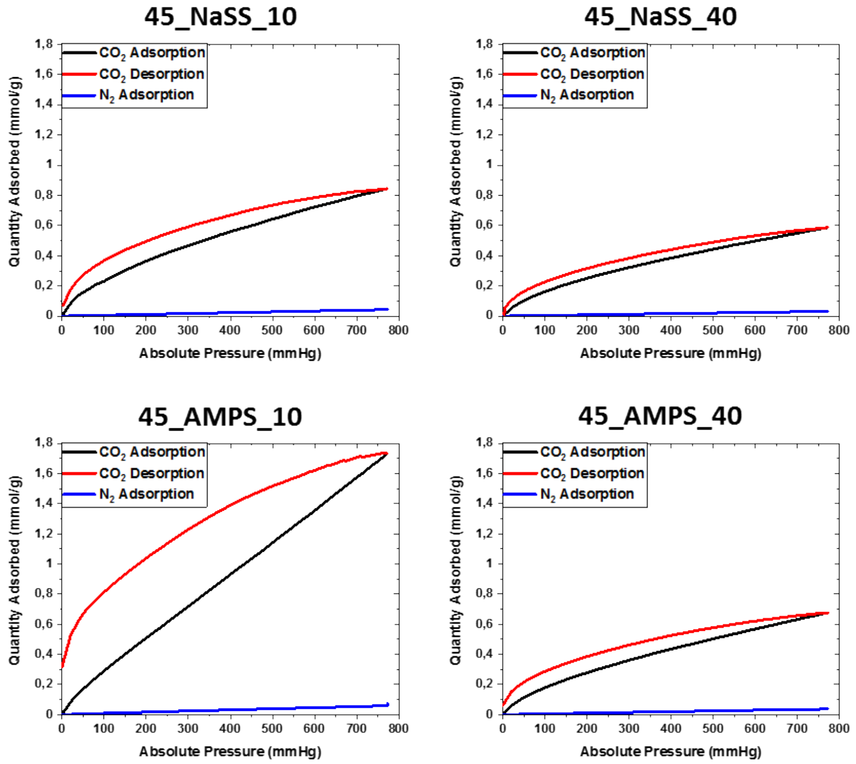

Figure S4. CO_2 adsorption-desorption and N_2 adsorption isotherms for composite aerogels containing 10 and 40% polymer, synthesized at 45 °C without pre-treatment.

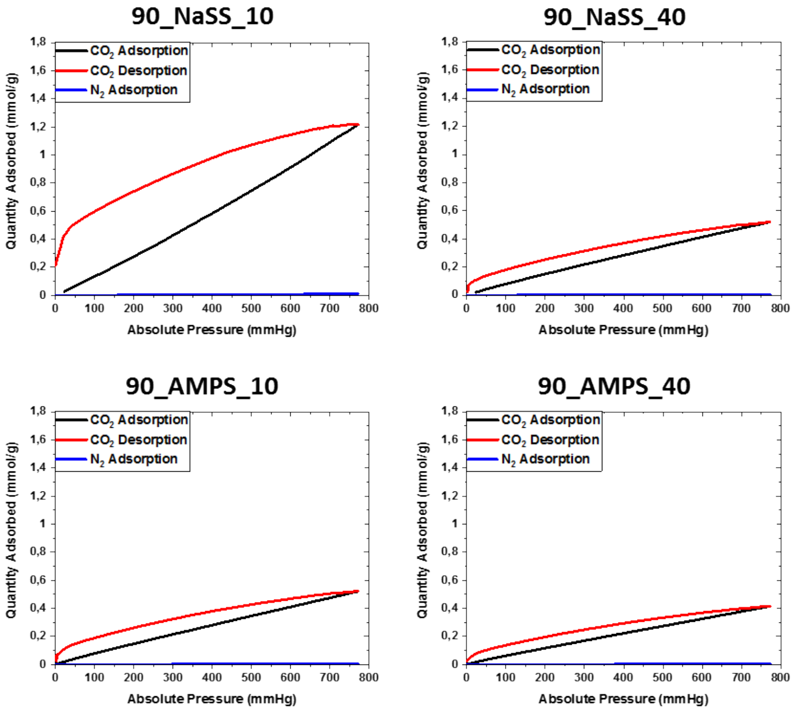

Figure S5. CO_2 adsorption-desorption and N_2 adsorption isotherms for composite aerogels containing 10 and 40% polymer, synthesized at 90 °C without pretreatment step.

Table S3. CO_2 and N_2 adsorption capacity and selectivity for composite aerogels. In brackets, the results of the previously reported 3D rGO-polymer structures synthesized by using the pre-treatment step are also presented

Material[a]	CO_2 adsorption (mmol g^{-1})	N_2 adsorption (mmol g^{-1})	Selectivity
**45_NaSS_10**	0.84 (0.67)	0.044 (0.076)	119 (51)
**45_NaSS_40**	0.59 (0.49)	0.032 (0.056)	113 (52)
**45_AMPS_10**	1.73 (0.67)	0.059 (0.072)	156 (53)
**45_AMPS_40**	0.68 (0.58)	0.038 (0.064)	108 (53)
**60_NaSS_10**	0.5	0.060	51
**60_NaSS_40**	0.43	0.063	42
**60_AMPS_10**	0.59	0.068	50
**60_AMPS_40**	0.49	0.065	52
**90_NaSS_10**	1.21 (0.63)	0.011 (0.069)	621 (49)
**90_NaSS_40**	0.52 (0.28)	0.006 (0.031)	471 (48)
**90_AMPS_10**	0.52 (0.28)	0.006 (0.033)	527 (45)
**90_AMPS_40**	0.42 (0.35)	0.005 (0.038)	496 (44)

[a]Nomenclature of the samples:
Reduction Tª_Type of F.M. (NaSS or AMPS) copolymerized with MMA_Weight % of the polymer (10 or 40)

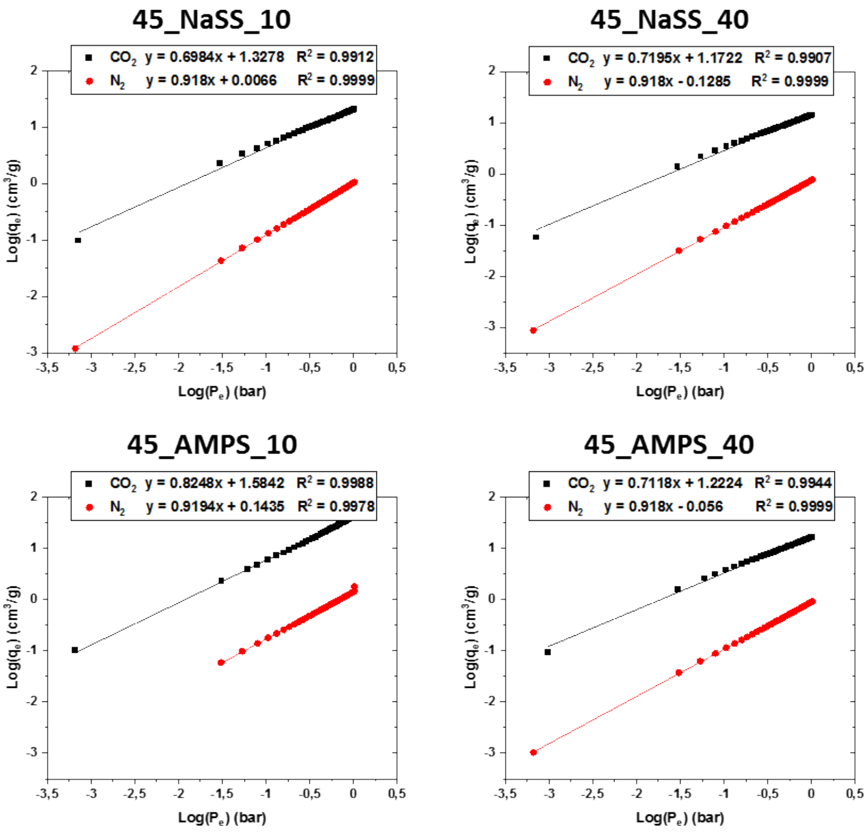

Figure S6. Freundlich equation fitting curve for CO_2 isotherm (black curve) and for N_2 isotherm (red curve) for composite aerogels synthesized at 45 °C without pretreatment.

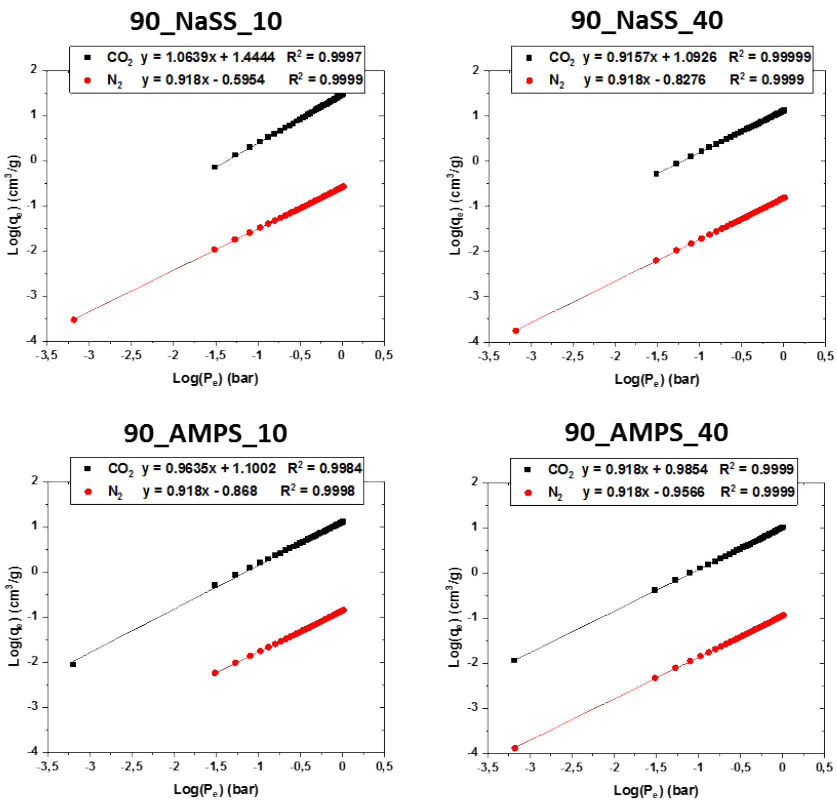

Figure S7. Freundlich equation fitting curve for CO_2 isotherm (black curve) and for N_2 isotherm (red curve) for composite aerogels synthesized at 90 °C without pretreatment.

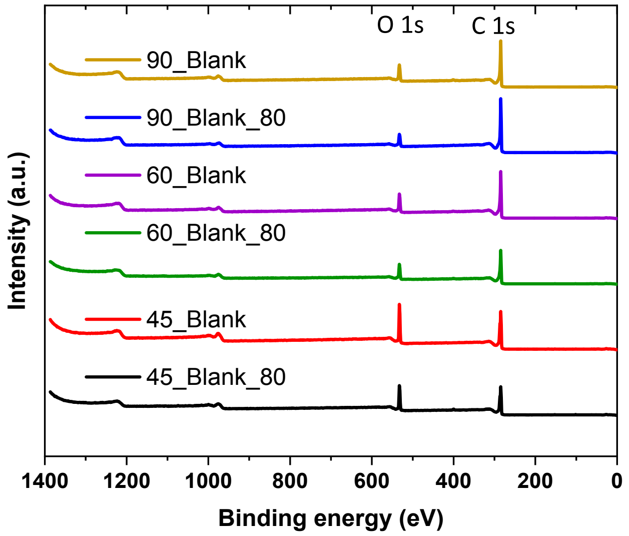

Figure S8. XPS survey spectra of 45_Blank_80, 45_Blank, 60_Blank_80, 60_Blank, 90_Blank_80, and 90_Blank aerogels.

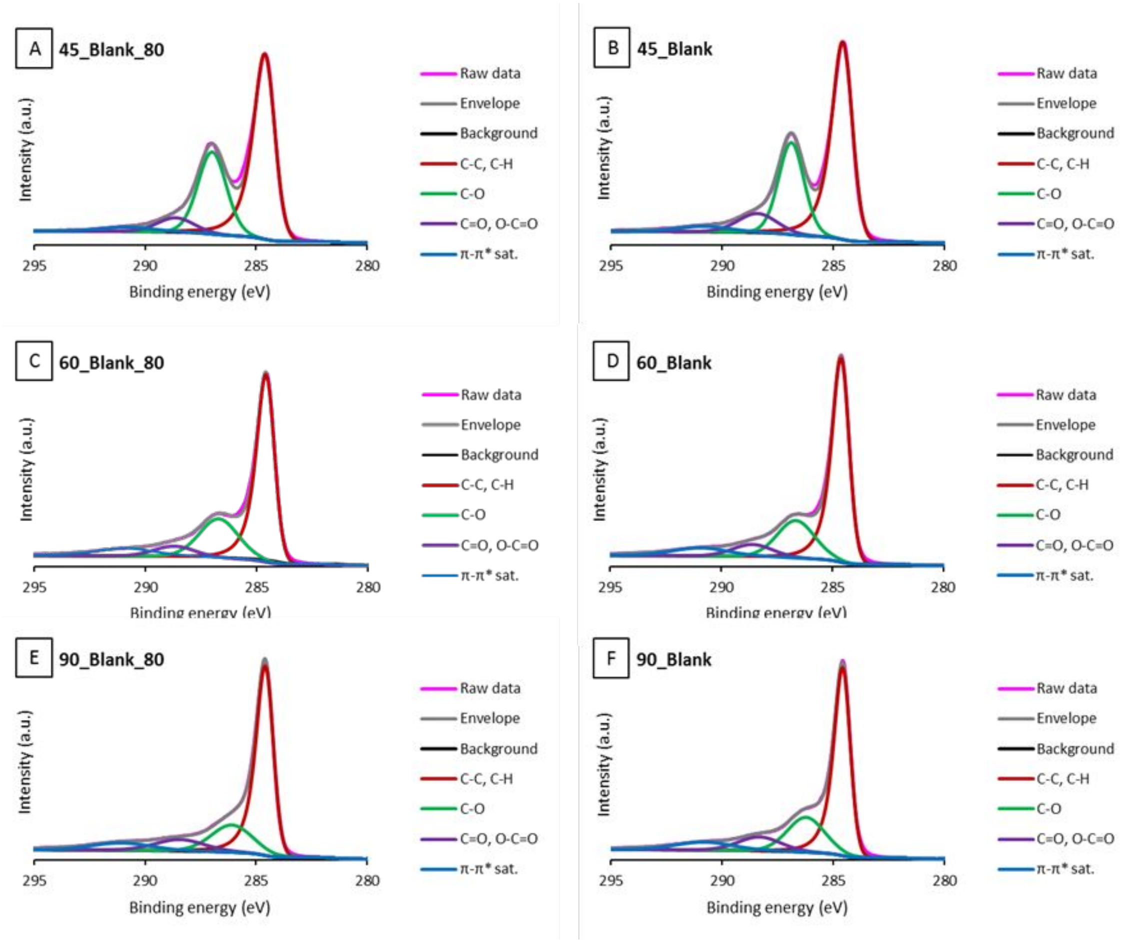

Figure S9. High-resolution C 1s XPS spectra of (A) 45_Blank_80, (B) 45_Blank, (C) 60_Blank_80, (D) 60_Blank, (E) 90_Blank_80, and (F) 90_Blank aerogels.
